# Novel Insect Antifeedant and Ixodicidal Nootkatone Derivatives

**DOI:** 10.3390/biom9110742

**Published:** 2019-11-16

**Authors:** Alberto Galisteo Pretel, Helena Pérez del Pulgar, A. Sonia Olmeda, Azucena Gonzalez-Coloma, Alejandro F. Barrero, José Francisco Quílez del Moral

**Affiliations:** 1 Department of Organic Chemistry, Institute of Biotechnology, University of Granada, 18071 Granada, Spain; albertogapre@ugr.es (A.G.P.); helenaperezpv@ugr.es (H.P.d.P.); 2 Faculty of Veterinary, Complutense University of Madrid (UCM), 28040 Madrid, Spain; angeles@ucm.es; 3 Institute of Agricultural Sciences, CSIC, 28006, Madrid, Spain; azu@ica.csic.es

**Keywords:** organic synthesis, natural products, pesticides, antiticks, antifeedant

## Abstract

Naturally occurring nootkatone, with reported insecticidal and acaricidal properties, has been used as a lead to generate molecular diversity and, consequently, new insect antifeedant and ixodicidal compounds. A total of 22 derivatives were generated by subjecting this molecule to several reactions including dehydrogenation with the iodine/DMSO system, oxidation with SeO_2_, epoxidation with *m*CPBA, oxidation or carbon homologations of the α-carbonyl position with TMSOTf (trimethylsilyl trifluoromethanesulfonate) followed by Rubottom and Dess Martin periodane oxidations, condensation with formaldehyde using Yb(OTf)_3_ as catalyst and dehydroxilation using the Grieco protocol. The insect antifeedant (against *Myzus persicae* and *Ropaloshysum padi*) and ixodicidal (against the tick *Hyalomma lusitanicum*) activities of these compounds were tested. Compound **20** was the most active substance against *M. persicae* and *R. padi*, and twice more efficient than nootkatone in the antitick test.

## 1. Introduction

The well-known advantages of biopesticides compared to conventional synthetic pesticides account for the increasing number of research papers published in this field in recent years [1,2]. Conventional pesticides and biopesticides can be categorized according to the origin of their active ingredients, with one of these categories encompassing synthetic natural derived (SND) substances [2]. In this context, we focused our attention on nootkatone (**1**), a sesquiterpene which was isolated for the first time from the essential oil of the heartwood of Alaska yellow cedar (*Cupressus nootkatensis*) [3,4], and is also present in plants of *Citrus* genus [5], *Vetiveria* species [6] and others natural sources [7]. Our interest in this molecule was based on its very interesting insecticidal and acaricidal properties [4,8,9,10,11,12,13,14]. In this sense, formulations acting as pesticides against mosquitoes and ticks are currently being developed [15]. However, the mode of action of this product remain uncertain [16].

Considering the repellent effects and insecticidal activity of nootkatone, we planned to use this renewable and chiral molecule as natural lead to generate molecular diversity with the ultimate goal of obtaining new SND insect antifeedants (against *Rhopalosiphum padi, Myzus persicae, Spodoptera littoralis*) and ixodicidal (against *Hyalomma lusitanicum*) compounds. The moth *S. littoralis*, also known as Egyptian cotton leaf worm, is reported to be a pest of this plant and of a high variety of vegetable crops [17]. The aphid *M. persicae* is known to infect many species from 40 plant families, with special prevalence in *Brassica* crops [18]. *R. padi* is also an aphid and is considered one of the most important cereal pest [19]. *Hyalomma lusitanicum* is a tick mainly found throughout the Mediterranean area. Apart from its widely known detrimental effects on several species of cattle, this pest is an efficient vector transmitter of the Crimean–Congo hemorrhagic fever disease [20].

**Figure 1:**
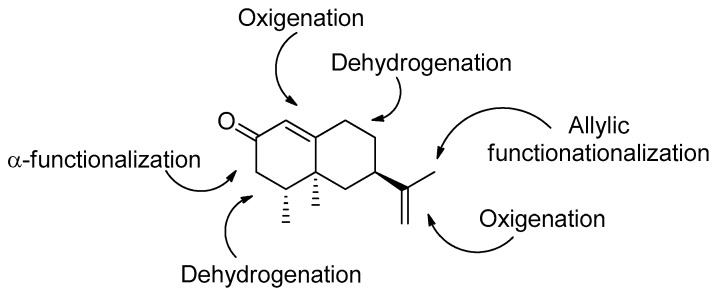
Generation of structural diversity from nootkatone (**1**).

Nootkatone features an appropriate structure with functionalizations that may allow an easy and efficient synthesis of a series of derivatives following a Diversity Oriented Synthesis (DOS) approach [21]. Thus, the presence of an unsaturated ketone would enable the production of further insaturations at C3–C4 or C8–C9. Also, the carbonyl group at C2 should permit the generation of new derivatives functionalized at C3. Additionally, the direct and allylic oxidations of the double bonds present in the molecule will help increase structural diversity (Figure 1).

To improve persistence of biological activity in open areas, new less volatile oxygenated nootkatone derivatives have been designed.

## 2. Experimental

### 2.1. Chemicals and Instrument

Silica gel 60 (35–70 μm) was used for flash column chromatography. NMR spectra (see Supplementary Materials) were obtained in a Varian Direct-Drive 600 (^1^H 600 MHz/^13^C 150 MHz), Varian Direct-Drive 500 (^1^H 500 MHz/^13^C 125 MHz) and Varian Direct-Drive 400 (^1^H 400 MHz/^13^C 100 MHz). Accurate mass determinations were performed on a SYNAPT G2-Si Q-TOF mass spectrometer (Waters, Milford, MA, USA) equipped with high-efficiency T-Wave ion mobility and an orthogonal Z–spray™ electrospray ionization (ESI) source. MassLynx v.4.1 software was used for HRMS instrument control, peak detection, and integration. Reactions were monitored by TLC on 0.25-mm E. Merck silica gel plates (60F-254) visualized under UV light and by applying a phosphomolybdic acid solution in EtOH followed by heat. High quality reagents were purchased at the highest quality that was commercially available and were used without further purification.

### 2.2. Synthesis of Nootkatone Derivatives (See Table 1, Scheme 1)

#### 2.2.1. Dehydrogenation of Nootkatone with the System I_2_/Dimethysulfoxide (DMSO)

To a solution of nootkatone (**1**), (219 mg, 1 mmol) in toluene (10 mL), I_2_ (25 mg, 0.1 mmol) and DMSO (0.34 mL, 5 mmol) were added under argon atmosphere. The resulting mixture was refluxed for 6 h. Upon consumption of the starting material, the reaction mixture was diluted with methyl *tert*-butyl ether (MTBE) (60 mL), washed with saturated Na_2_S_2_O_4_ (3 × 30 mL), brine (3 × 30 mL), dried over anhydrous Na_2_SO_4_, and concentrated in vacuo. The crude product was purified by flash chromatography (H:MTBE 4:1) to obtain **2 [22]** (31 mg, 14%), **3** [23] (110 mg, 51%) and **4** (56 mg, 16%).

**Compound 4**: [α]_D_ = +34.3 (*c* = 1, DCM). ^1^H NMR (600 MHz CDCl_3_) δ 6.23 (dd, *J* = 10.3, 2.3 Hz, 1H), 6.20 (d, *J* = 10.3 Hz, 1H), 5.84 (s, 1H), 4.94 (d, *J* = 12.5 Hz, 1H), 4.87 (s, 1H), 4.82 (s, 1H), 3.03 (dd, *J* = 11.3, 4.6 Hz, 1H), 2.28 (dq, *J* = 13.2, 6.7 Hz, 1H), 2.03 (dd, *J* = 12.9, 4.9 Hz, 1H), 1.75 (s, 3H), 1.33 (t, *J* = 12.2 Hz, 1H), 1.27 (d, *J* = 6.7 Hz, 3H), 1.08 (s, 3H). ^13^C NMR (151 MHz, CDCl_3_) δ 192.88 (C), 161.97 (C), 146.81 (C), 141.63 (CH), 127.53 (CH), 121.74 (CH), 112.01 (CH_2_), 48.70 (CH), 41.80 (CH), 41.67 (CH), 39.44 (CH_2_), 38.71 (C), 20.47 (CH_3_), 17.62 (CH_3_), 16.99 (CH_3_). HRMS TOF (ESI+) *m*/*z* calculated for C_15_H_19_IO [M + H]^+^ 342.0475, found 342.0481.

#### 2.2.2. Dehydrogenation of **1** with 2,3-dichloro-5,6-dicyanobenzoquinone (DDQ)

To a solution of nootkatone **1** (655 mg, 3 mmol) in 20 mL of benzene, under an argon atmosphere, was added DDQ (749 mg, 3.3 mmol) and 0.22 mL of acid acetic. The reaction mixture was stirred for 2 days in reflux. Then, the solution was filtered at room temperature and the solvent was evaporated under low pressure. The crude reaction mixture was then re-dissolved in EtOAc, and washed with saturated NaHCO_3_ and brine, dried with anhydrous Na_2_SO_4_, and concentrated in vacuo. Purification by flash chromatography (H:MTBE, 1:1) provided 328 mg of **3** [23] (51%) and 103 mg of **1** (16%).

#### 2.2.3. Reaction of Nootkatone with SeO_2_ (See Scheme 2)

To a solution of nootkatone **1** (216 mg, 0.99 mmol) in EtOH (10 mL), SeO_2_ (100 mg, 1 mmol) was added and the resulting solution was refluxed for 3 h. Upon consumption of the starting material, the reaction mixture was concentrated in vacuo. The crude reaction mixture was solubilized in EtOAc (50 mL), washed with saturated NaHCO_3_ (3 × 30 mL), brine (3 × 30 mL), dried over anhydrous Na_2_SO_4_, and concentrated in vacuo. Purification by flash chromatography was carried out (H:MTBE, from 4:1 to 1:1) to give 75 mg of **5** [24] (32%), 28 mg of **6** [24] (11%) and 35 mg of **7** (15%).

**Compound 7**. [α]_D_= −0.39 (*c* = 1, DCM). ^1^H NMR (500 MHz, CDCl_3_) δ 4.94 (s, 1H), 4.80 (s, 1H), 2.69 (d, *J* = 16.3 Hz, 1H), 2.56 (d, *J* = 16.4 Hz, 1H), 2.26–2.12 (m, 4H), 1.77 (s, 3H), 1.72–1.77 (m, 3H), 1.54–1.45 (m, 2H), 1.11 (d, *J* = 0.8 Hz, 3H), 0.90 (d, *J* = 5.8 Hz, 3H). ^13^C NMR (126 MHz, CDCl_3_) δ = 209.05 (C), 145.62 (C), 109.61 (CH_2_), 89.89 (C), 88.66 (C), 49.73 (CH_2_), 45.91 (C), 44.66 (CH_2_), 44.35 (CH_2_), 37.96 (CH), 35.92 (CH_2_), 32.85 (CH_2_), 18.91 (CH_3_), 15.33 (CH_3_), 14.27 (CH_3_). HRMS TOF (ESI+) *m*/*z* calculated for C_15_H_23_O_2_ [M + H]^+^ 235.1691, found 235.1698.

#### 2.2.4. Acetylation of Compound **5**

To a solution of **5** (300 mg, 1.28 mmol) in pyridine (10 mL), 3 mg of 4-dimethhylaminopyridine were added under argon atmosphere and the resulting mixture was heated to reflux temperature. Then Ac_2_O (1.82 mL, 19.2 mmol) was added and the mixture refluxed for 1 h. Upon consumption of the starting material, 10 g of ice and 50 mL of MTBE were poured over the crude mixture. The organic layer was washed with 2N HCl (3 × 30 mL), saturated NaHCO_3_ (3 × 30 mL) and brine (3 × 30 mL), dried over anhydrous Na_2_SO_4_, and concentrated in vacuo. The crude product was purified by flash chromatography (H:MTBE, 1:2) to give 291 mg of **8** (82%).

**Compound 8**. [α]_D_= +99.8 (*c* = 1, DCM). ^1^H NMR (400 MHz, CDCl_3_) δ 5.75 (s, 1H), 5.07 (s, 1H), 4.96 (s, 1H), 4.56 (s, 2H), 2.50 (dddd, *J* = 15.6, 13.7, 5.1, 2.0 Hz, 1H), 2.43–2.29 (m, 2H), 2.29–2.16 (m, 2H), 2.08 (s, 3H), 2.06–1.87 (m, 3H), 1.35 (dd, *J* = 13.6, 12.6, 4.2 Hz, 1H), 1.25–1.10 (m, 1H), 1.09 (s, 3H), 0.94 (d, *J* = 6.8 Hz, 3H).^13^C NMR (101 MHz, CDCl_3_) δ = 199.35 (C), 170.64 (C), 169.63 (C), 147.34 (C), 124.83 (CH_2_), 111.83 (CH), 66.01 (CH_2_), 44.15 (CH_2_), 42.00 (CH_2_), 40.36 (CH), 39.33 (C), 36.37 (CH), 32.90 (CH_2_), 31.74 (CH_2_), 20.93 (CH_3_), 16.70 (CH_3_), 14.85(CH_3_). HRMS TOF (ES+) *m*/*z* calculated for C_17_H_25_O_3_ [M + H]+ 277.1804, found 277.1831.

#### 2.2.5. Reaction of Compound **3** with SeO_2_ (See Scheme 3)

To a solution of **3** (115 mg, 0.53 mmol) in EtOH (5 mL), SeO_2_ (96 mg, 0.86 mmol) was added and the resulting solution was the refluxed for 3 h. The reaction mixture was then concentrated in vacuo, re-dissolved in EtOAc (30 mL), washed with saturated NaHCO_3_ (3 × 20 mL), brine (3 × 20 mL), dried over anhydrous Na_2_SO_4_, and concentrated in vacuo. The crude product was purified by flash chromatography (H:MTBE, from 1:2 to 1:4) to obtain 30 mg of **3** (26%), 65 mg of **9** (53%) and 17 mg of **10** (14%).

**Compound 9**. [α]_D_= +29.6 (*c* = 1, DCM). ^1^H NMR (500 MHz, CDCl_3_) δ = 6.11 (s, 2H), 5.10 (s, 1H), 4.90 (s, 1H), 4.16 (s, 2H), 2.71–2.54 (m, 2H), 2.50 (dddd, *J* = 15.6, 13.7, 5.1, 2.0 Hz, 1H), 2.18–2.03 (m, 2H), 2.01 (s, 3H), 1.47–1.36 (m, 1H), 1.36 (s, 3H), 1.28 (t, *J* = 12.8 Hz, 1H). ^13^C NMR (126 MHz, CDCl3) δ = 186.39 (C), 167.10 (C), 165.66 (C), 151.57 (C), 126.78 (CH), 124.61 (CH), 109.82 (CH_2_), 65.30 (CH_2_), 43.46 (C), 42.40 (CH_2_), 35.72 (CH), 33.90 (CH_2_), 32.85 (CH_2_), 23.24 (CH_3_), 18.86 (CH_3_). HRMS TOF (ESI+) *m*/*z* calculated for C_15_H_21_O_2_ [M + H]^+^ 233.1542, found 233.1671.

**Compound 10**. [α]_D_= +40.6 (*c* = 1, DCM). ^1^H NMR (500 MHz, CD_3_COCD_3_) δ 9.59 (s, 1H), 6.42 (d, *J* = 1.1 Hz, 1H), 6.18 (s, 1H), 6.00 (dt, *J* = 4.8, 1.7 Hz, 2H), 3.12 (ttd, *J* = 12.7, 3.5, 1.1 Hz, 1H), 2.77 (ddd, *J* = 13.8, 4.9, 1.7 Hz, 1H), 2.46 (ddd, *J* = 13.9, 4.2, 2.6 Hz, 1H), 2.16 (ddd, *J* = 12.7, 3.5, 2.3 Hz, 1H), 2.05–1.99 (m, 4H), 1.44 (s, 3H), 1.43–1.37 (m, 1H), 1.26 (t, *J* = 12.7 Hz, 1H). ^13^C NMR (126 MHz, CD_3_COCD_3_) δ 194.04 (CH), 184.78 (C), 166.14 (C), 164.92 (C), 153.28 (C), 133.45 (CH_2_), 126.35 (CH), 124.30 (CH), 43.25 (C), 41.72 (CH_2_), 33.16 (CH_2_), 32.23 (CH_2_), 30.77 (CH), 22.66 (CH_3_), 17.89 (CH_3_). HRMS TOF (ESI+) *m*/*z* calculated for C_15_H_19_O_2_ [M + H]^+^ 231.1382, found 231.1385.

#### 2.2.6. Reaction of Nootkatone with *m*chloroperbenzoic Acid (*m*CPBA) (See Scheme 4)

To a solution of nootkatone (**1**) (220 mg, 1 mmol) in DCM (6 mL), *m*CPBA (595 mg, 3.4 mmol) was added and stirred at room temperature for 5 days. Upon consumption of the starting material, the reaction mixture was diluted with MTBE (50 mL), washed with 10% Na_2_SO_3_ (3 × 30 mL), 10% NaOH (3 × 30 mL) and brine (3 × 30 mL), dried over anhydrous Na_2_SO_4_, and concentrated in vacuo. The crude product was purified by flash chromatography (H:MTBE, 3:1) to obtain 49 mg of **11** [25] (21%), 58 mg of **12 [26]** (23%) and 79 mg of **13** (30%).

**Compound 13**. [α]_D_= +5.5 (*c* = 1, DCM). ^1^H NMR (400 MHz, CDCl_3_) δ 4.78 (s, 2H), 2.89 (m, 2H), 2.64–2.52 (m, 4H), 2.43–2.37 (m, 2H), 1.92–1.74 (m, 8H), 1.67–1.44 (m, 2H), 1.38–1.15 (m, 6H), 1.25 (s, 3H), 1.24 (s, 3H), 1.08 (s, 3H), 1.08 (s, 3H), 0.99 (d, *J* = 7.2, Hz, 3H), 0.98 (d, *J* = 7.2, Hz, 3H). ^13^C NMR (101 MHz, CDCl_3_) δ = 170.35, 170.31 (C), 86.80, 86.68 (CH), 69.13, 69.05 (C), 58.76, 58.59 (C), 53.50, 52.87 (CH_2_), 40.60, 40.37 (CH_2_), 38.65 (CH_2_), 38.37, 37.87 (CH), 38.23 (C), 37.64, 37.62 (CH), 30.78, 30.61 (CH_2_), 26.21, 25.73 (CH_2_), 18.54, 17.92 (CH_3_), 17.12, 17.07 (CH_3_), 16.60, 16.58 (CH_3_). HRMS TOF (ES+) *m*/*z* calculated for C_15_H_23_O_4_ [M + H]^+^ 267.1583, found 267.1596.

#### 2.2.7. Synthesis of Compounds **14** and **15** (See Scheme 5)

To a solution of nootkatone **1** (0.46 mL, 2 mmol) in dry toluene (9 mL) at −78 °C, 2.5 mL of collidine were added and stirred for 15 min, then TMSOTf (0.9 mL, 5 mmol) was added and the resulting solution was stirred for additional 10 min. Upon consumption of the starting material, the reaction mixture was diluted with hexane (15 mL), washed with brine (3 × 10 mL) and dried over anhydrous Na_2_SO_4_ and concentrated in vacuo. The crude product was solved in dry DCM (30 mL) at 0 °C and then NaHCO_3_ (672 mg, 8 mmol) and *m*CPBA (592 mg, 2,4 mmol) were added. The resulting mixture was stirred for 3 h. The reaction mixture was then diluted with DCM (5 mL) filtered through Celite® and concentrated in vacuo. The crude product was purified using mixtures of eluents of increasing polarity to obtain 30 mg of **1** (7%) (H:MTBE 9:1), 76 mg of **14** (16%) (H:MTBE, 5:1) and 77 mg of **15** (16%) (H:MTBE,5:1).

**Compound 14**. [α]_D_= +137.8 (*c* = 1, DCM). ^1^H NMR (600 MHz, CDCl_3_) δ = 5.85 (s, 1H), 4.74 (s, 1H), 4.70 (s, 1H), 3.96 (d, *J* = 12.7 Hz, 1H), 2.52 (td, *J* = 13.7, 4.5 Hz, 1H), 2.40 (ddd, *J* = 14.9, 4.2, 2.6 Hz, 1H), 2.34 (tt, *J* = 12.5, 3.3 Hz, 1H), 1.97 (dt, *J* = 13.1, 2.9 Hz, 1H), 1.91–1.95 (m, 1H), 1.82 (dq, *J* = 13.2, 6.7 Hz, 1H), 1.72 (s, 3H), 1.36 (qd, *J* = 12.9, 4.2 Hz, 1H), 1.22 (s, 3H), 1.14 (d, *J* = 6.7 Hz, 3H). ^13^C NMR (151 MHz, CDCl_3_) δ = 199.65 (C), 172.08 (C), 148.63 (C), 120.98 (CH), 109.46 (CH_2_), 73.40 (CH), 47.63 (CH), 44.18 (CH_2_), 40.92 (C), 39.61 (CH), 33.14 (CH_2_), 31.75 (CH_2_), 20.75 (CH_3_), 17.79 (CH_3_), 10.81 (CH_3_). HRMS TOF (ESI+) *m*/*z* calculated for C_15_H_23_O_2_ [M + H]^+^ 235.1689, found 235.1698.

**Compound 15**. [α]_D_= -49.8 (*c* = 1, DCM). ^1^H NMR (400 MHz, CDCl_3_) δ = 5.82 (s, 1H), 4.72 (s, 1H), 4.70 (s, 1H), 4.36 (d, *J* = 5.2 Hz, 1H), 2.62–2.46 (m, 2H), 2.27 (ddd, *J* = 12.7, 4.3, 2.7 Hz, 1H), 2.07–1.96 (m, 2H), 1.83–1.71 (m, 2H), 1.70 (s, 3H), 1.37–1.28 (m, 1H), 1.26 (s, 3H), 0.91 (d, *J* = 7.0 Hz, 3H).^13^C NMR (151 MHz, CDCl_3_) δ = 199.59 (C), 170.58 (C), 148.13 (C), 119.46 (CH), 109.69 (CH_2_), 73.23 (CH), 43.87 (CH), 43.58 (CH_2_), 41.11 (C), 39.91 (CH), 34.37 (CH_2_), 32.98 (CH_2_), 22.54 (CH_3_), 20.93 (CH_3_), 8.86 (CH_3_). HRMS TOF (ESI+) *m*/*z* calculated for C_15_H_23_O_2_ [M + H]^+^ 235.1689, found 235.1709.

#### 2.2.8. Synthesis of Compounds **16** and **17**

To a solution of the compounds **14** and **15** (40 mg, 0.17 mmol) dry DCM (1 mL), DMP (89 mg, 0.21 mmol) was added under argon atmosphere. The mixture was stirred for 90 min. Then, a saturated solution of Na_2_S_2_O_3_-NaHCO_3_ (3 mL) was added and the resulting mixture was stirred for 30 additional min. Upon consumption of the starting material, the reaction mixture was diluted in MTBE (10 mL), washed with NaHCO_3_ (3 × 7 mL) and brine (3 × 7 mL), dried with anhydrous Na_2_SO_4_, and concentrated in vacuo. Purification by flash chromatography (H:TMBE, 3:1) gave 10 mg of **16** (23%) and 6 mg of **17** (14%).

**Compound 16**. [α]_D_= −10.4 (*c* = 1, DCM). ^1^H NMR (400 MHz, CDCl_3_) δ = 6.47 (s, 1H), 6.22 (s, 1H), 4.76 (s, 1H), 4.74 (s, 1H), 2.63 (td, *J* = 13.8, 5.0 Hz, 1H), 2.51–2.41 (m, 2H), 2.11 (dt, *J* = 13.0, 3.0 Hz, 1H), 2.08–2.01 (m, 1H), 1.97 (s, 3H), 1.72 (s, 3H), 1.41–1.35 (m, 1H), 1.34 (s, 3H), 1.20 (t, *J* = 12.8 Hz, 1H). ^13^C NMR (126 MHz, CDCl_3_) δ = 180.44 (C), 170.50 (C), 147.92 (C), 143.85 (C), 134.29 (C), 121.51 (CH), 110.01 (CH_2_), 43.50 (C), 42.53 (CH_2_), 39.87 (CH), 33.63 (CH_2_), 32.98 (CH_2_), 23.220 (CH_3_), 20.66 (CH_3_), 10.81 (CH_3_). HRMS TOF (ES+) *m*/*z* calculated for C_15_H_21_O_2_ [M + H]^+^ 233.1542, found 233.1531.

**Compound 17**. [α]_D_= +105.2 (*c* = 1, DCM). ^1^H NMR (400 MHz, CDCl_3_) δ = 6.19 (s, 1H), 4.79 (s, 1H), 4.77 (s, 1H), 2.63 (dddd, *J* = 15.2, 13.3, 5.1, 1.9 Hz, 1H), 2.55 (ddd, *J* = 14.9, 4.4, 2.6 Hz, 1H), 2.41 (tt, *J* = 12.6, 3.5 Hz, 1H), 2.06–1.97 (m, 1H), 1.89 (t, *J* = 12.9 Hz, 1H), 1.84–1.77 (m, 1H), 1.76 (s, 3H), 1.54–1.43 (m, 1H), 1.42 (s, 3H), 1.29 (s, 3H).^13^C NMR (126 MHz, CDCl_3_) δ = 197.15 (C), 186.11 (C), 172.62 (C), 148.08 (C), 124.92 (CH), 110.03 (CH_2_), 81.64 (C), 48.43 (C), 39.41 (CH), 37.48 (CH_2_), 33.69 (CH_2_), 31.89 (CH_2_), 22.59 (CH_3_), 20.74 (CH_3_), 17.11 (CH_3_). HRMS TOF (ESI+) *m*/*z* calculated for C_15_H_21_O_3_ [M + H]^+^ 249.1491, found 249.1483.

#### 2.2.9. Synthesis of Compounds **18** and **19** (See Scheme 6)

To a solution of nootkatone **1** (0.23 mL, 1 mmol) in dry toluene (4 mL) cooled at −78 °C, 1.25 mL of collidine were added and the resulting mixture was stirred for 15 min, then TMSOTf (0.45 mL, 2.5 mmol) was added and stirred for 10 additional min. Upon consumption of the starting material, the reaction mixture was diluted with hexane (15 mL), washed with brine (3 × 10 mL) and dried over anhydrous Na2SO4 and concentrated in vacuo. The crude mixture was dissolved in THF (10 mL), then 4 mL of formaldehyde (37%, water) and Yb(OTf)_3_ (550 mg, 0.9 mmol) were added under argon atmosphere and stirred for 24 h. The reaction mixture was then diluted with a solution of saturated NaHCO_3_ (60 mL) and extracted with EtOAc (3 × 15 mL). The organic layer was washed with 1N HCl (3 × 15 mL), brine (3 × 15 mL), dried with anhydrous Na_2_SO_4_ and concentrated in vacuo. The crude reaction mixture was purified by column chromatography using mixtures of solvents with increasing polarity to obtain 126 mg of **1** (54.7%) (H:MTBE, 9:1), 86 mg of **18** (34%) (H:MTBE, 2:1).and 30 mg of **19** (11%) (H:MTBE, 2:1).

**Compound 18**. [α]_D_= +118.0 (*c* = 1, DCM). ^1^H NMR (400 MHz, CDCl_3_) δ = 5.77 (d, *J* = 1.6 Hz, 1H), 4.73 (bs, 1H), 4.70 (bs, 1H), 4.04 (dd, *J* = 11.2, 3.1 Hz, 1H), 3.72 (dd, *J* = 11.4, 6.7 Hz, 1H), 2.49 (dddd, *J* = 15.6, 14.0, 5.2, 1.9 Hz, 1H), 2.38–2.28 (m, 3H), 2.00 (dt, *J* = 13.1, 2.8 Hz, 1H), 1.94–1.88 (m, 2H), 1.71 (s, 3H), 1.33 (qd, *J* = 12.7, 4.1 Hz, 1H), 1.14 (s, 3H), 1.11 (t, *J* = 13.0 Hz, 1H), 0.99 (d, *J* = 6.8 Hz, 3H).^13^C NMR (126 MHz, CDCl_3_) δ = 202.50 (C), 170.84 (C), 148.86 (C), 124.17 (CH), 109.39 (CH_2_), 60.39 (CH_2_), 49.18 (CH), 44.04 (CH_2_), 41.27 (CH), 40.26 (CH), 39.79 (C), 33.05 (CH_2_), 31.60 (CH_2_), 20.73 (CH_3_), 17.25 (CH_3_), 12.12 (CH_3_). HRMS TOF (ESI+) *m*/*z* calculated for C_16_H_25_O_2_ [M + H]^+^ 249.1855, found 249.1843.

**Compound 19**. [α]_D_= +238.7 (*c* = 1, DCM). ^1^H NMR (400 MHz, CDCl_3_) δ 5.54 (dd, *J* = 5.2, 2.3 Hz, 1H), 4.77 (bs, 1H), 4.74 (bs, 1H), 4.04 (d, *J* = 11.7 Hz, 1H), 3.99 (d, *J* = 11.5 Hz, 1H), 3.93 (d, *J* = 11.5 Hz, 1H), 3.72 (d, *J* = 11.7 Hz, 1H), 2.79 (dd, *J* = 18.6, 7.8 Hz, 1H), 2.34–2.05 (m, 4H), 1.95 (ddd, *J* = 17.6, 11.2, 2.3 Hz, 1H), 1.75 (s, 3H), 1.75–1.70 (m, 1H), 1.22 (7, *J* = 12.5 Hz, 1H), 0.95 (d, *J* = 6.6 Hz, 3H), 0.86 (s, 3H).^13^C NMR (126 MHz, CDCl_3_) δ 218.09 (C), 149.25 (C), 142.18 (C), 122.99 (CH), 109.09 (CH_2_), 70.60 (2 x CH_2_), 57.81 (C), 44.22 (CH_2_), 41.14 (CH_2_), 38.08 (C), 37.28 (CH), 36.62 (CH), 31.18 (CH_2_), 20.71 (CH_3_), 17.82 (CH_3_), 15.26 (CH_3_). HRMS TOF (ESI+) *m*/*z* calculated for C_17_H_27_O_3_ [M + H]^+^ 279.1960, found 279.1950.

#### 2.2.10. Synthesis of Compound **20**

To a solution of **18** (75 mg, 0.3 mmol) in dry TFH (2.5 mL), 0.13 mL of *n*-Bu_3_P (0.46 mmol) and 1.3 mg of *o*-NO_2_PhSeCN (0.46 mmol) were added under argon atmosphere and the resulting solution was stirred for 80 min at room temperature. Upon consumption of starting material, the reaction mixture was diluted with MTBE (10 mL), was washed with saturated NH_4_Cl (3 × 5 mL), brine (3 × 5 mL), dried with anhydrous Na_2_SO_4_, and concentrated in vacuo. The crude reaction mixture was dissolved in THF (30 ml) and 30% H_2_O_2_ (0.06 mL, 0.48 mmol) was added under argon atmosphere. The resulting mixture was stirred for 15 min at room temperature. Upon consumption of the starting material, the reaction mixture was diluted with MTBE (30 mL), washed with brine (3 x 20 mL), dried with anhydrous Na_2_SO_4_, and concentrated in vacuo and purified by flash chromatography (H: MTBE, 1:2) to obtain 48 mg of **20** (71%).

**Compound 20.** [α]_D_= +149.2 (*c* = 1, DCM). ^1^H NMR (400 MHz, CD_3_COCD_3_) δ 5.92 (s, 1H), 5.80 (s, 1H), 5.22 (s, 1H), 4.75 (bs, 1H), 4.73 (bs, 1H), 2.67–2.53 (m, 2H), 2.44 (ddd, *J* = 15.3, 4.1, 2.8 Hz, 1H), 2.37 (tt, *J* = 12.6, 3.3 Hz, 1H), 2.03–1.98 (m, 1H), 1.95–1.87 (m, 1H), 1.75 (s, 3H), 1.42–1.25 (m, 2H), 1.14 (d, *J* = 6.7, 0.9 Hz, 3H), 1.02 (s, 3H). ^13^C NMR (101 MHz, CD_3_COCD_3_) δ 187.79 (C), 170.28 (C), 149.29 (C), 147.31 (C), 124.13 (CH), 116.87 (CH_2_), 108.59 (CH_2_), 46.01 (CH), 43.75 (CH_2_), 40.95 (C), 40.25 (CH), 32.57 (CH_2_), 31.31 (CH_2_), 20.08 (CH_3_), 17.74 (CH_3_), 9.70 (CH_3_). HRMS TOF (ESI+) *m*/*z* calculated for C_16_H_23_O [M + H]^+^ 231.1749, found 231.1739.

#### 2.2.11. Synthesis of Compounds **21** and **22** (See Scheme 7)

To solution of **8** (290 mg, 1.05 mmol) in dry toluene (5 mL) cooled at −78 °C, 1.5 mL of collidine was added under argon atmosphere and the resulting mixture was stirred for 15 min at room temperature, then 0.47 mL of TMSOTf (2.5 mmol) were added and stirred for another 10 min. The reaction mixture was diluted in hexane (20 mL) and was washed with brine (3 × 15 mL), dried with anhydrous Na_2_SO_4_, and concentrated in vacuo. The crude mixture was dissolved in dry THF (10 mL), and then 5 mL of formaldehyde (37% water solution) and 1 g of Yb(OTf)_3_ (1.6 mmol) were added under argon atmosphere and stirred for 24 h at room temperature. Upon consumption of the starting material, the reaction mixture was diluted with 60 mL of saturated NaHCO_3_ solution and then was extracted with EtOAc (3 × 35 mL). The combined organic layers were washed with 1N HCl (3 × 20 mL), and brine (3 × 20 mL), dried with anhydrous Na_2_SO_4_, and concentrated in vacuo. The crude product was purified by flash chromatography (H:MTBE, 1:3) to obtain 187 mg of **8** (64.5%) 62 mg of **21** (19.3%) and 22 mg of **22** (6.9%).

**Compound 21**. [α]_D_= +111.2 (*c* = 1, DCM). ^1^H NMR (400 MHz, CDCl_3_) δ 5.79 (s, 1H), 5.09 (s, 1H), 4.97 (s, 1H), 4.60 (d, *J* = 13.6 Hz, 1H), 4.56 (d, *J* = 13.3 Hz, 1H), 4.06 (dd, *J* = 11.4, 3.1 Hz, 1H), 3.73 (dd, *J* = 11.4, 6.6 Hz, 1H), 2.86 (sa, 1H), 2.52 (dddd, *J* = 15.5, 13.8, 5.1, 1.9 Hz, 1H), 2.43–2.28 (m, 3H), 2.10 (s, 3H), 2.09–2.05 (m, 1H), 2.02–1.89 (m, 2H), 1.37 (qd, *J* = 12.8, 4.2 Hz, 1H), 1.15 (s, 3H), 1.14–1.09 (m, 1H), 1.00 (d, *J* = 6.8 Hz, 3H). ^13^C NMR (126 MHz, CDCl_3_) δ = 202.27 (C), 170.66 (C), 170.05 (C), 147.21 (C), 124.35 (CH), 112.03 (CH_2_), 66.03 (CH_2_), 60.25 (CH_2_), 49.19 (CH), 44.27 (CH_2_), 41.16 (CH), 39.83 (C), 36.29 (CH), 32.97 (CH_2_), 31.77 (CH_2_), 20.96 (CH_3_), 17.14 (CH_3_), 12.12 (CH_3_). HRMS TOF (ESI+) *m*/*z* calculated for C_18_H_27_O_4_ [M + H]^+^ 307.1909, found 307.1950.

**Compound 22**. [α]_D_= +18.1 (*c* = 1, DCM). ^1^H NMR (400 MHz, CDCl_3_) δ 5.84 (s, 1H), 5.11 (s, 1H), 4.99 (s, 1H), 4.63-4.55 (m, 2H), 3.90 (dd, *J* = 10.8, 9.6 Hz, 1H), 3.72 (dd, *J* = 10.8, 3.9 Hz, 1H), 2.69–2.60 (m, 1H), 2.56–2.47 (m, 2H), 2.36 (ddd, *J* = 13.5, 4.3, 2.7 Hz, 1H), 2.11 (s, 3H), 2.10–2.02 (m, 2H), 1.89 (dt, *J* = 13.0, 3.1 Hz, 1H), 1.45 (t, *J* = 12.6 Hz, 1H), 1.43–1.32 (m, 1H), 1.15 (s, 3H), 1.00 (d, *J* = 7.1 Hz, 3H). ^13^C NMR (126 MHz, CDCl_3_) δ 202.64 (C), 170.67 (C), 170.05 (C), 146.85 (C), 122.97 (CH), 112.15 (CH_2_), 66.06 (CH_2_), 61.07 (CH_2_), 52.39 (CH), 44.42 (CH_2_), 41.58 (CH), 39.97 (C), 36.15 (CH), 33.36 (CH_2_), 33.02 (CH_2_), 21.01 (CH_3_), 20.96 (CH_3_), 11.47 (CH_3_). HRMS TOF (ESI+) *m*/*z* calculated for C_18_H_27_O_4_ [M + H]^+^ 307.1909, found 307.1925.

#### 2.2.12. Synthesis of Compound **23** (See Scheme 7)

A solution of **21** (20 mg, 0.06 mmol) was stirred in 0.5 mL deuterated chloroform (HCl: 0.01%) for 7 days at 4 °C. Then, the solvent was evaporated, and the crude mixture was purified by flash chromatography (H:MTBE, 1:3) to obtain 11 mg of **23** (66.7%) and 6 mg of **21** (30%).

**Compound 23**: [α]_D_= +99.8 (*c* = 1, DCM). ^1^H NMR (600 MHz, CDCl_3_) δ 6.07 (s, 1H), 5.89 (s, 1H), 5.25 (s, 1H), 5.10 (s, 1H), 4.99 (s, 1H), 4.60 (d, *J* = 13.5 Hz, 1H), 4.57 (d, *J* = 13.3 Hz, 1H), 2.62–2.7 (m, 1H), 2.51–2.44 (m, 2H), 2.37–2.31 (m, 1H), 2.10 (s, 3H), 2.03 (dt, *J* = 13.1, 2.8 Hz, 1H), 1.99–1.93 (m, 1H), 1.40 (qd, *J* = 12.8, 4.6 Hz, 1H), 1.25 (t, *J* = 12.9 Hz, 1H), 1.12 (d, *J* = 6.8 Hz, 3H), 0.99 (s, 3H). ^13^C NMR (151 MHz, CDCl_3_) δ 189.41 (C), 170.68 (C), 170.20 (C), 147.34 (C), 146.45 (C), 124.81 (CH), 118.74 (CH_2_), 111.93 (CH_2_), 66.02 (CH_2_), 45.96 (CH), 44.05 (CH_2_), 41.19 (C), 36.53 (CH), 32.99 (CH_2_), 29.67 (CH_2_), 20.95 (CH_3_), 18.24 (CH_3_), 10.32 (CH_3_). HRMS TOF (ESI+) *m*/*z* calculated for C_18_H_25_O_3_ [M + H]^+^ 289.1804, found 289.1836.

### 2.3. Antifeedant Activity

Insect colonies maintained at ICA-CSIC were used for conducting the bioassays. *M. persicae* and *R. padi* were reared on bell pepper (*Capsicum annuum*) and *Hordeum vulgare* plants, respectively, and maintained at 22 ± 1 °C and >70% relative humidity, with a photoperiod of 16:8 h (L:D) in a growth chamber.

Antifeedant bioassays: The upper surface of *C. anuum* and *H. vulgare* leaf disks or fragments (1.0 cm^2^) were treated with 10 μl of the test substance. The products were tested at an initial dose of 5 mg/ml (50 µg/cm^2^) respectively. Twenty ventilated plastic boxes (2 × 2 cm) with 10 apterous aphid adults (24–48 h old) each were allowed to feed in a growth chamber (24 h, environmental conditions as above). Each experiment was repeated 2 (SE < 10%) and terminated after 24 h. Aphid settling was measured by counting the number of aphids on each leaf fragment. Settling inhibition (%FI or %SI) was calculated as % FI / %SI = [1 − (T/C) × 100], where T and C represent settling on treated and control leaf disks, respectively. The antifeedant effects (% SI) were analyzed for significance by the nonparametric Wilcoxon signed-rank test. Extracts and compounds with an SI > 70% were further tested in a dose-response experiment (3–4 serial dilutions) to calculate their relative potency (EC50, the effective dose to give a 50% settling reduction) from linear regression analysis (% FI/SI on Log-dose) [27].

### 2.4. Ixodicidal Activity

*H. lusitanicum* engorged female ticks were collected in central Spain (Finca La Garganta, Ciudad Real) from their host (deer) and maintained at 22–24 °C and 70% RH until oviposition and egg hatching. Resulting larvae (4–6 weeks old) were used for the bioassays [12]. Briefly, 50 µL of test solution were added to 25 mg of powdered cellulose at different concentrations (initial concentration of 10 mg/ml) and the solvent was evaporated. For each test, three replicates with 20 larvae each were used. Dead ticks were counted after 24 h of contact with the treated cellulose at the environmental conditions described, using a binocular magnifying glass. The larvicidal activity data are presented as percent mortality corrected according to Schneider–Orelli’s formula. Effective lethal doses (LC_50_ and LC_90_) were calculated by Probit Analysis (5 serial dilutions, STATGRAPHICS Centurion XVI, version 16.1.02, Statgraphics Technologies, Inc., P.O. Box 134, The Plains, Virginia 20198, USA).

## 3. Results and Discussion

### 3.1. Synthesis of Nootkatone Derivatives

The generation of chemical diversity from nootkatone (**1**) started by subjecting this molecule to the iodine/DMSO dehydrogenating system [28,29]. The reaction of **1** with 0.1 equiv of I_2_ and 5 equiv of DMSO in refluxing toluene led to the production of three dehydrogenated products **2**–**4** (Table 1, entry 1). Under these conditions, dienone **3** was obtained as the major compound [23]. Compound **2** is a natural component of grapefruit juice [22]. Compound **4** is described for the first time here. Its stereochemistry at C3 was assigned based on the proton J value (*J* = 12.5 Hz) of the proton at this position.

**Scheme 1:**
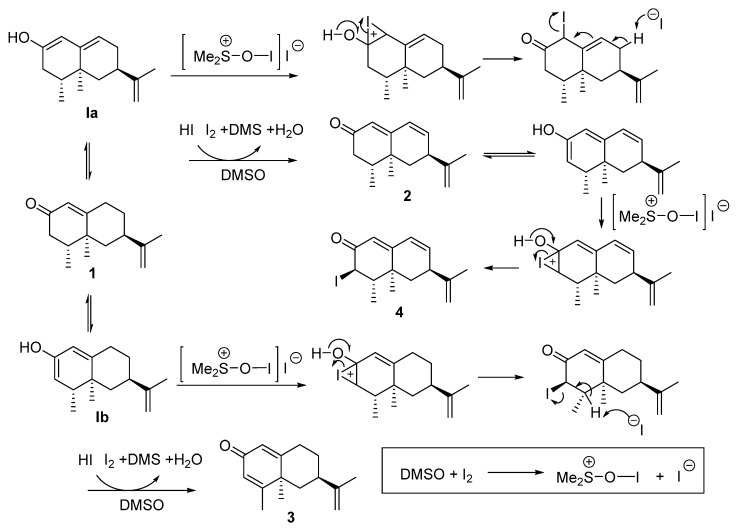
Proposed mechanism for the generation of **2**–**4**.

When 0.2 mmol of I_2_ were used (Table 1, entry 2), the same mixture of compounds was obtained, with compound **2** being the main reaction product (50% yield). When the quantity of I_2_ increased to 1 mmol (Table 1, entry 3), a different proportion of the same mixture **2**–**4** was obtained, with compounds **2** (28%) and **4** (25%) being then the main reaction products. A mechanistic proposal for the formation of these substances is shown in Scheme 1. Thus, when **1** reacts via **Ia**, compounds **2** and **4** are obtained, whereas if **Ib** is the reacting species, the process leads to the generation of **3**.

The regioselective dehydrogenation of nootkatone also afforded dienone **3** (62%) after its treatment with DDQ/AcOH in benzene.

**Scheme 2:**
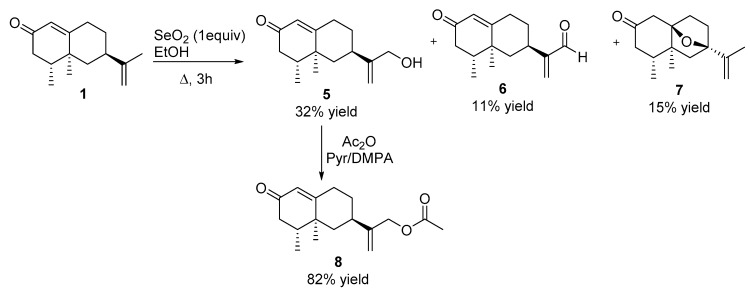
Synthesis of nootkatone derivatives **5**–**8**.

The oxidation of **1** with SeO_2_ in refluxing EtOH for 3 h led to compounds **5**–**7** (Scheme 2), with alcohol **5** being the major product. Compound **7** was formed after allylic oxidation at C7 and subsequent Michael addition of this hydroxyl group to C10. Alcohol **5** is a natural product isolated from *Alpinia oxyphylla* [30], and it was also found in P450-catalyzed transformations of nootkatone [31]. Compound **6** was previously synthesized from nootkatone by a photochemical process [24].

**Figure 2:**
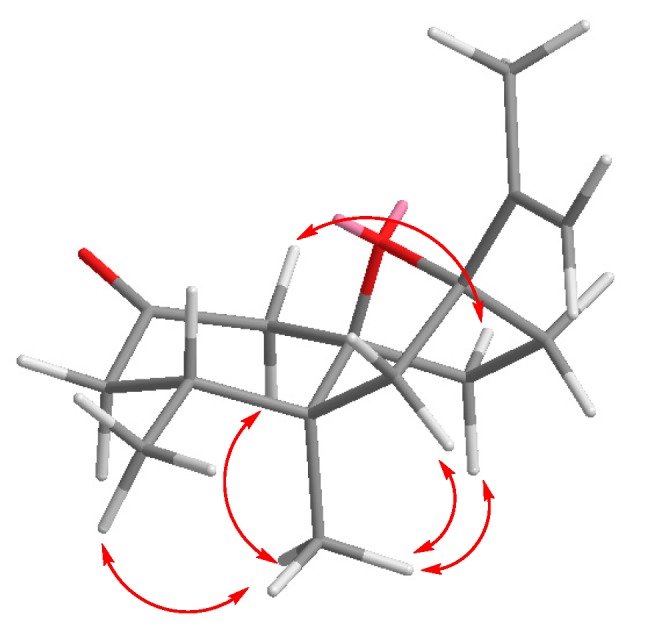
Selected NOEs observed for compound **7**.

Due to the overlapping signals observed for compound **7** in its ^1^H-NMR spectrum, the configurations at C17 and C10 could only be only assigned after analysis of the corresponding stepNOESY experiments (selective TOCSY edited preparation NOESY experiences) [32] (Figure 2).

**Scheme 3:**
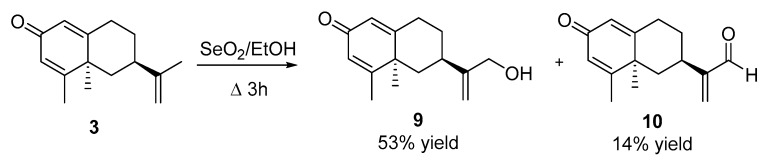
Synthesis of nootkatone derivatives **9**–**10**.

Oxidation of dienone **3** with SeO_2_ under the same experimental conditions gave **9**–**10** with acceptable yields (Scheme 3).

**Scheme 4:**
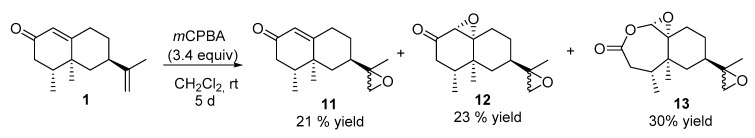
Synthesis of nootkatone derivatives **11**–**12**.

Epoxidation of **1** with *m*CPBA at 0 °C for 3 h afforded mainly monoepoxide **11** [33]. With longer reaction times reaction times, excess of *m*CPBA and at room temperature, the mixture of epoxides **11** and **12 [26]** was obtained along with diepoxide **13**, generated via the Baeyer–Villiger oxidation of the C1–C2 bond of diepoxide **12** (Scheme 4).

**Scheme 5:**
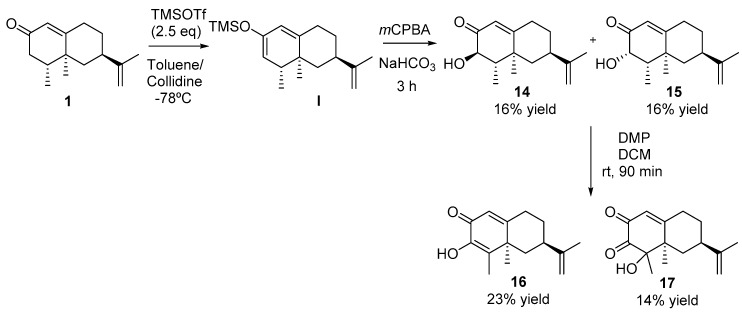
Synthesis of nootkatone derivatives **14**–**17**.

Then the reactivity of the α-carbonyl methylene of nootkatone to trigger either oxidations or carbon homologations was addressed. The incorporation of a hydroxyl group at C3 was achieved via treatment of **1** with TMSOTf (trimethylsilyl trifluoromethanesulfonate) and collidine to produce the TMS-dienol intermediate **I**, which after a Rubottom oxidation [34] evolved towards the corresponding α-hydroxy carbonyl stereoisomers **14**–**15**. These compounds were easily oxidized with the Dess Martin periodane to afford the dicarbonyl derivatives **16**–**17** (Scheme 5).

**Figure 3:**
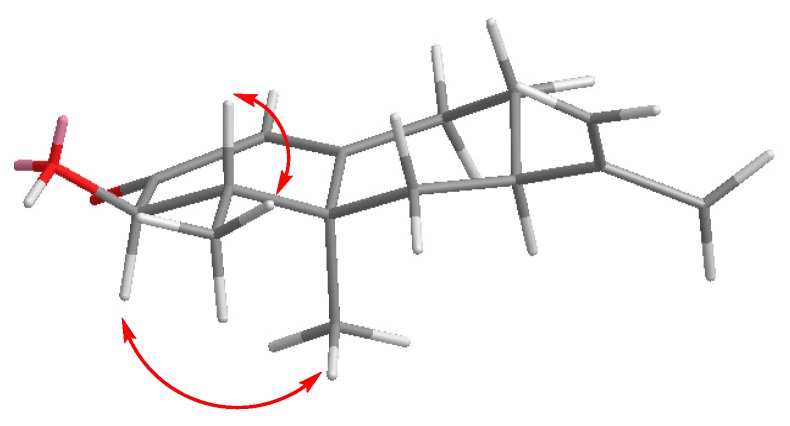
Selected NOESY correlations observed for compound **14**.

As happened with 4, the stereochemistry at C3 of alcohols 14 and 15 was determined on the basis of the coupling constant values measured for H3 (14: H3, d, *J* = 12.7; 15: H3, d *J* = 5.2): Additionally, the correlations observed in the 2D NOESY spectrum of 14 confirmed this assignment (Figure 3).

**Scheme 6:**
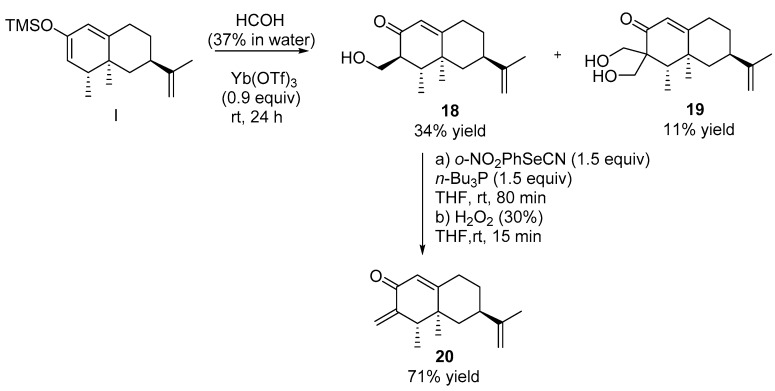
Synthesis of nootkatone derivatives **18**–**20**.

Additionally, the same TMS-dienol **I** was condensated with formaldehyde (37% in water) using Yb(OTf)_3_ as catalyst [35]. Under these conditions aldol **18** was obtained in a 34% yield together with minor proportions of compound **19** (11%), generated a result of a double condensation process. Starting from **18**, the elimination of the hydroxyl group was achieved in two steps following the Grieco protocol [36] to obtain a good yield of dienone **20** in good (71%) (Scheme 6).

**Figure 4:**
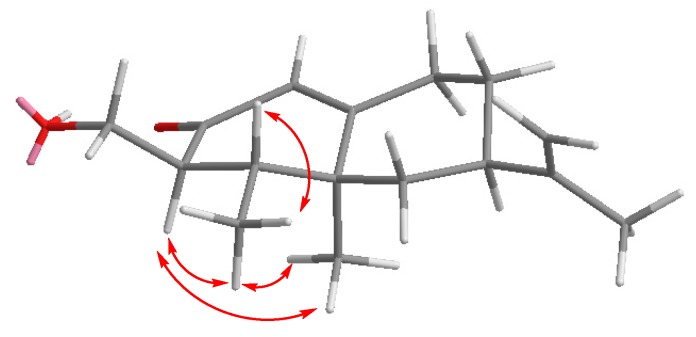
Selected NOEs observed for compound **18.**

The C3 stereochemistry of alcohol **18** was determined following analysis of the NOEs observed in the selective 1D-NOESY spectra of this compound (Figure 4).

**Scheme 7:**
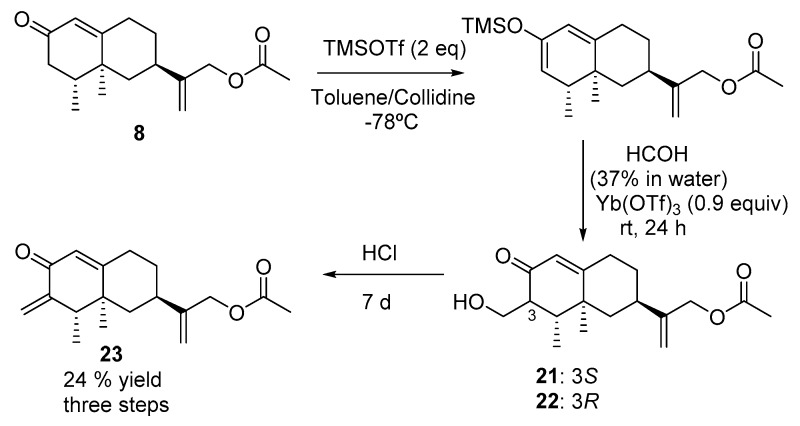
Synthesis of nootkatone derivatives **21**–**23**.

Following a similar sequence, the acetoxy derivative **23** was synthesized from **8** in three steps (Scheme 7).

**Figure 5:**
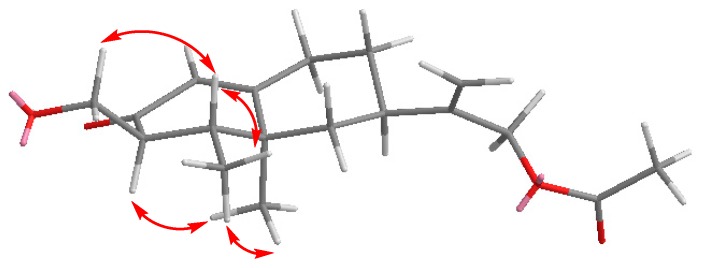
Selected NOEs observed for compound **21.**

The enhancements observed in the selective 1D NOESY of **21** allowed us to assign the stereochemistry of this compound at C3 (Figure 5).

### 3.2. Antifeedant Activity

**Figure 6:**
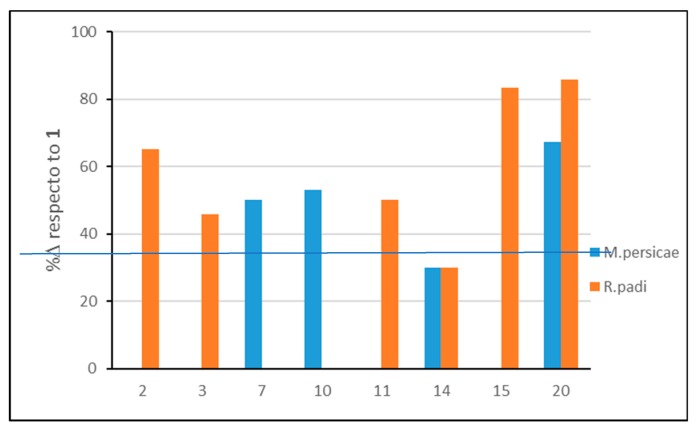
Increment (%Δ) in antifeedant activity (EC_50_ values) of derivatives **2**, **3**, **7**, **10**, **11**, **14**, **15**, and **20** with respect to **1**.

Table 2 shows the results of the aphid antifeedant effects of nootkatone (**1**) and derivatives. These compounds were also tested against the lepidopteran *S. littoralis* without significant effects (all EC_50_ > 50). The most active compounds were **20** (both species), **15** and **2** (*R. padi*) followed by **7**, **10** (*M. persicae*), **3** (*R. padi*) and **14** (both species). All these derivatives were more active than nootkatone (**1**), with **20**, **15** and **2** being over 50% more potent, followed by **10**, **7**, and **11** (~50%) (Figure 6).

It should be noticed that the α,β-unsaturated ketone **20,** with an extra methylene, **15** with an α-hydroxy at C-3 and **2,** with an additional endocyclic C8–C9 unsaturation, exhibited strong antifeedant effects followed by the exocyclic epoxide **7**, and the aldehyde derivative **10**. The presence of an additional Michael acceptor in compound **20** may account for the observed bioactivity of this product. In this regard, previous results showed that the presence of an exocyclic epoxide in eremophilanes resulted in strong antifeedant effects against *M. persicae* and *S. littoralis* [12].

### 3.3. Ixodicidal Activity

Table 3 shows the ixodicidal effects of nootkatone (**1**) and derivatives against *H. lusitanicum* larvae.

**Figure 7:**
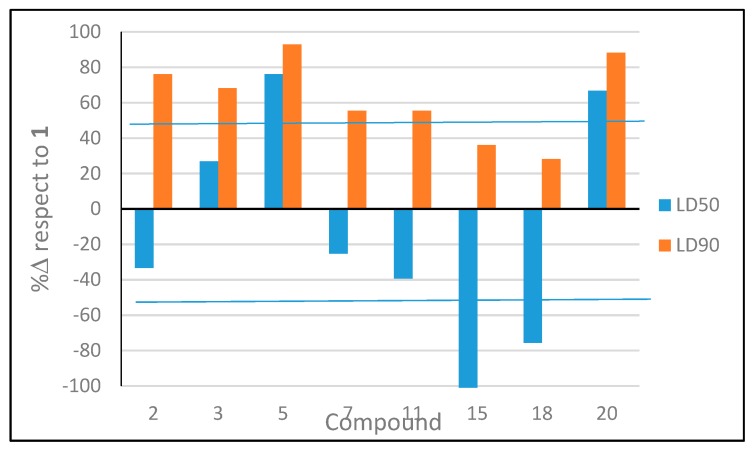
Increment (%Δ) in ixodicidal activity (LD_50_ and LD_90_ values) of derivatives **2**, **3**, **5**, **7**, **11**, **15**, **18**, and **20** respect to **1**.

Using the activity of nootkatone as reference, the tested compounds can be classified in four groups according to their relative activity (Figure 7). Compounds **5**, **20** (>50%) and **3** (20 and 68%) showed an increase in acaricidal activity with respect to nootkatone (**1**) for both lethal doses (Table 3, Figure 5); followed by **2, 7** and **11** with an increase for LD_90_ >50%. Compounds **15** and **18** showed reduced acaricidal potency at LD_50_ and higher at LD_90_ than **1**. Lastly, the remaining compounds either displayed a significant loss of activity or were not active at all. These results are relevant considering that nootkatone was reported to be more active against the tick species *Ixodes scapularis* and *Amblyomma americanum* than Permanone, a permethrin-based commercial clothing repellent [37].

From these data, the following structure–activity relationships were inferred for the acaricidal effects. Hydroxylation at C13 (compound **5**) increased acaricidal activity, possibly due to a decrease in the volatility of the molecule. The presence of a methylene group at C3 (compound **20**) also increased activity with respect to nootkatone, most likely due to the presence of a more accessible Michael acceptor. Also, an additional endocyclic unsaturation increased the ixodicidal effects (C3–C4 in **3** and C8–C9 in **2**). Finally, it can be concluded that the unsaturated ketone present in the structure of **1** plays a key role in the antitick activity of the tested substances whereas the C11–C12 double bond enhances the antitick efficiency as previously shown [12].

## 4. Conclusions

Starting from the natural product nootkatone (**1**), a known biopesticide compound, we have synthesized 22 derivatives in a reduced number of steps. Two are known natural products (**2** and **5**) and 15 have been synthesized for the first time. With these derivatives, we performed antifeedant test against *S. littoralis, M. persicae* and *R. padi*, and ixodicidal tests against *H. lusitanicum*. Compounds **20**, **14** and **2** were more antifeedant (>50% more potent) than **1**, followed by **7**, **10** and **11** (~50%). Compounds **5**, **20**, and **3** were stronger acaricidal agents than **1** for both lethal doses, followed by **2, 7** and **11** (LD_90_ >50%). Compound **20** was the only compound with stronger effects than **1** on both targets (insects and ticks). In this regard, the introduction of an additional methylene moiety at C3 enhanced antifeedant and ixodicidal activities. The significance of these results is supported by the fact that nootkatone is subject to research and development to support US Environmental Protection Agency registration for commercial marketing as a repellent and biopesticide against mosquitoes and ticks [38].

## Figures and Tables

**Table 1:** Reaction of nootkatone with the dehydrogenating system I_2_/DMSO.

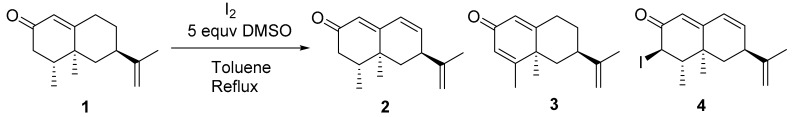					
Entry	Iodine (equiv)	Time (h)	2	3	4
1	0.1	30	14	51	16
2	0.2	6	50	28	–
3	1	2	28	6	25

**Table 2:** Insect antifeedant effects of nootkatone (**1**) and derivatives **2**–**23** against aphids.

Product	*M. persicae*		*R. padi*	
	50 (µg /cm^2^) ^a^	EC_50_^b^	50 (µg /cm^2^) ^a^	EC_50_^b^
**1**	50.7 ± 9.1	~50	57.7 ± 10.2	~50
**2**	35.4 ± 9.0	>50	86.0 ± 3.6 *	17.4 (14.4–20.9)
**3**	24.6 ± 7.5	>50	77.1 ± 4.5 *	27.1 (20.7–35.4)
**5**	49.4 ± 10.9	~50	40.9 ± 8.2	>50
**6**	57.2 ± 10.1	~50	30.7 ± 6.8	>50
**7**	79.9 ± 5.4 *	~25	30.8 ± 6.5	>50
**8**	37.8 ± 9.8	>50	31.4 ± 5.9	>50
**9**	34.2 ± 8.5	>50	44.2 ± 9.2	>50
**10**	80.6 ± 4.8 *	23.5 (16.6–33.3)	36.95 ± 8.5	>50
**11**	49.0 ± 9.4	~50	69.4 ± 7.6 *	~25
**12**	59.1 ± 7.0	~50	51.8 ± 5.7	~50
**13**	29.0 ± 4.5	>50	19.85 ± 6.5	>50
**14**	91.2 ± 5.3 *	30–40	83.8 ± 4.9 *	30–40>50
**15**	54.5 ± 8.0	~50	78.81 ± 4.4	8.3 (5.5–12.7)
**16**	63.9 ± 7.4 *	~50	37.3 ± 7.3	>50
**17**	40.6 ± 6.9	>50	34.2 ± 7.9	>50
**18**	60.4 ± 7.1 *	~50	44.4 ± 9.0	>50
**19**	35.4 ± 7.6	>50	19.8 ± 5.6	>50
**20**	92.1± 2.5 **	16.3 (13.6–19.7)	93.9 ± 4.2 *	7.1 (5.4–9.4)
**23**	62.3 ± 9.2	~50	17.5 ± 6.7	>50

^a^ Percent settling inhibition (%SI, n = 100 insects). ^b^ Dose needed to give 50% settling inhibition (95% Confidence Limits). * Significantly different from the control (*p* < 0.05), Wilcoxon paired rank test.

**Table 3:** Acaricidal activity of nootkatone (**1**) and derivatives **2**–**23** against *H. lusitanicum* larvae.

Product	% Mortality ^a^ (20 μg/mg)	Lethal Dose (μg/mg) ^b^	
		LD_50_	LD_90_
**1**	85.9 ± 2.2	4.02 (1.92–7.42)	18.02 (13.60–29.16)
**2**	100 ± 0	5.36 (4.94–5.90)	4.31(3.93–4.82)
**3**	98.3 ± 0.4	2.94 (2.54–3.38)	5.72 (5.0–6.80)
**5**	100 ± 0	0.96 (0.88–1.04)	1.28 (1.18–1.38)
**6**	47.3 ± 14.3		
**7**	100 ± 0	5.04 (4.56–5.64)	8.04 (7.20–9.22)
**8**	32.2 ± 3.0		
**9**	42.6 ± 2.2		
**10**	8.5 ± 1.0		
**11**	88.5 ± 1.6	5.60 (4.88–6.44)	10.84 (9.50–12.8)
**12**	1.5 ± 1.6		
**13**	7.7 ± 10.8		
**14**	46.3 ± 8.7		
**15**	100 ± 0	8.74 (8.10–9.40)	11.52 (10.68–12.8)
**16**	23.4 ± 3.5		
**17**	11.7 ± 7.3		
**18**	96.7 ± 1.6	7.06 (6.20–8.10)	12.94 (11.34–15.36)
**19**	17.7 ± 1.3		
**20**	100 ± 0	1.34 (1.20–1.50)	2.14 (1.92–2.44)
**23**	11.5 ± 1.3		

^a^ Mortality data corrected according to Schneider–Orelli’s formula. ^b^ Doses needed to give 50 and 90% mortality (95% Confidence Limits).

## Supplementary Materials

The supplementary material related to this article includes NMR spectra of new compounds and is available online at https://www.mdpi.com/2218-273X/9/11/742/s1.
