# Supplementary material for: Novel Insect Antifeedant and Ixodicidal Nootkatone Derivatives
Source: Biomolecules. 2019 Nov 16;9(11):742. doi: 10.3390/biom9110742 (PMC6921050; doi:10.3390/biom9110742)
Supplement: Supplementary file 1 [file biomolecules-09-00742-s001.pdf]

Novel insect antifeedant and ixodicidal nootkatone derivatives

Alberto Galisteo Pretel[1], Helena Pérez del Pulgar[1], A. Sonia Olmeda[2], Azucena Gonzalez-Coloma[3], Alejandro F. Barrero[1,*] and José Francisco Quílez del Moral[1,*]

[1] Department of Organic Chemistry, Institute of Biotechnology, University of Granada, 18071 Granada, Spain; albertogapre@ugr.es (A.G.); helenaperezpv@ugr.es (H.P.P.)

[2] Faculty of Veterinary, Complutense University of Madrid (UCM), 28040 Madrid, Spain; angeles@ucm.es (A.S.O.)

[3] Institute of Agricultural Sciences, CSIC, 28006, Madrid, Spain; azu@ica.csic.es (A.G.C.)

* Correspondence: afbarre@ugr.es; jfquilez@ugr.es; Tel.: +34-958243185

List of contents

NMR spectra 2-24

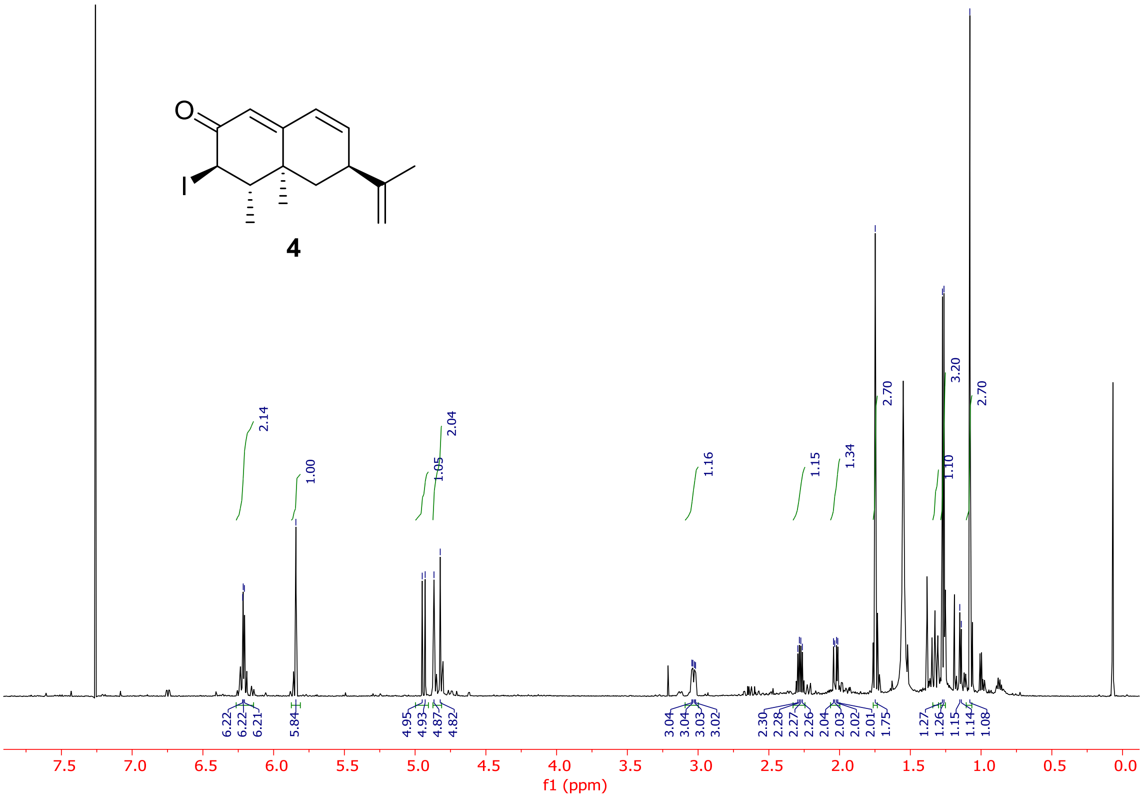
O
I
4
2.14
1.00
1.05
2.04
1.16
1.15
1.34
2.70
1.10
3.20
2.70
6.22
6.22
6.21
5.84
4.95
4.93
4.87
4.82
3.04
3.04
3.03
3.02
2.30
2.28
2.27
2.26
2.04
2.03
2.02
2.01
1.75
1.27
1.26
1.15
1.14
1.08
7.5 7.0 6.5 6.0 5.5 5.0 4.5 4.0 3.5 3.0 2.5 2.0 1.5 1.0 0.5 0.0
f1 (ppm)

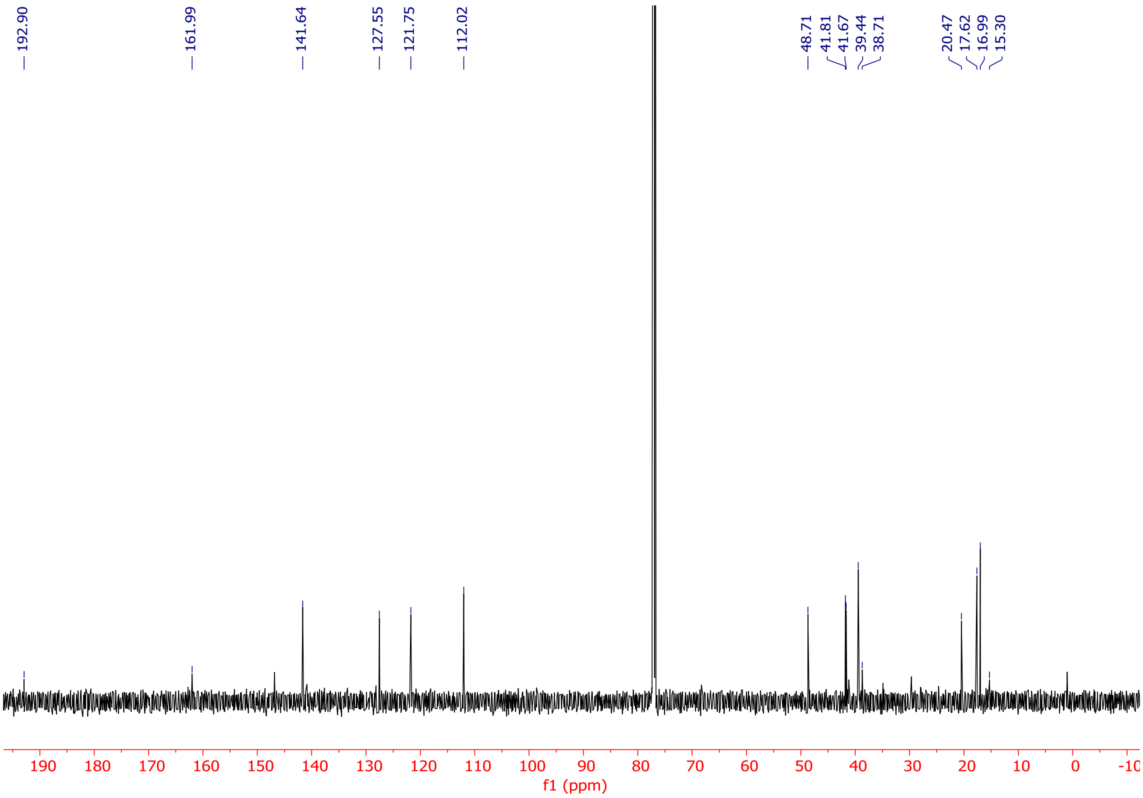
192.90
161.99
141.64
127.55
121.75
112.02
48.71
41.81
41.67
39.44
38.71
20.47
17.62
16.99
15.30
190 180 170 160 150 140 130 120 110 100 90 80 70 60 50 40 30 20 10 0 -10
f1 (ppm)

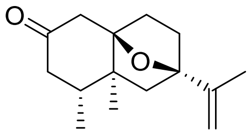
O
O

7

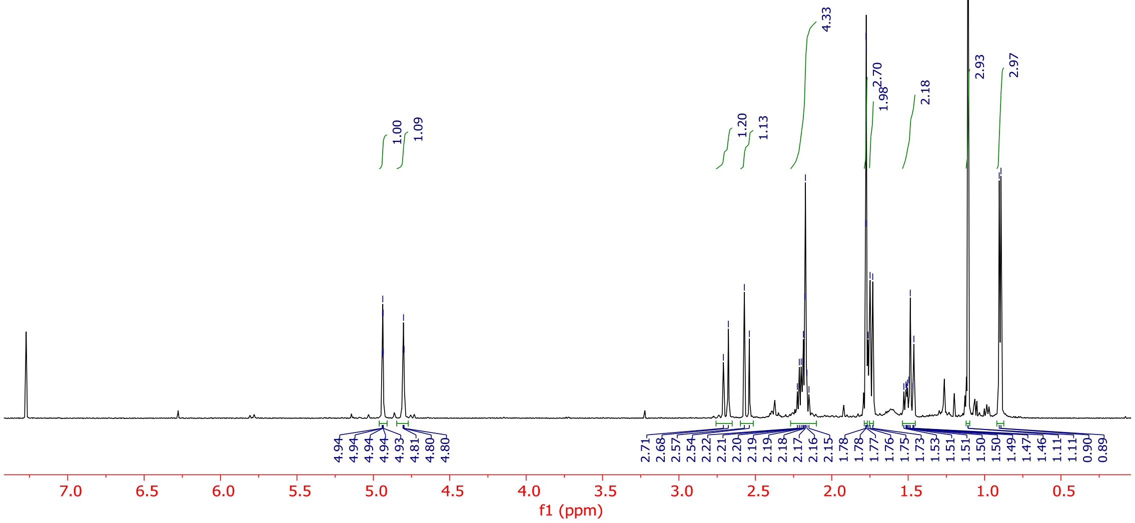
1.00
1.09
1.20
1.13
4.33
2.70
1.98
2.18
2.93
2.97
4.94
4.94
4.94
4.94
4.93
4.81
4.80
4.80
2.71
2.68
2.57
2.54
2.22
2.21
2.20
2.19
2.19
2.18
2.17
2.16
2.15
1.78
1.77
1.76
1.75
1.73
1.53
1.51
1.51
1.50
1.50
1.49
1.47
1.46
1.11
1.11
0.90
0.89
7.0 6.5 6.0 5.5 5.0 4.5 4.0 3.5 3.0 2.5 2.0 1.5 1.0 0.5
f1 (ppm)

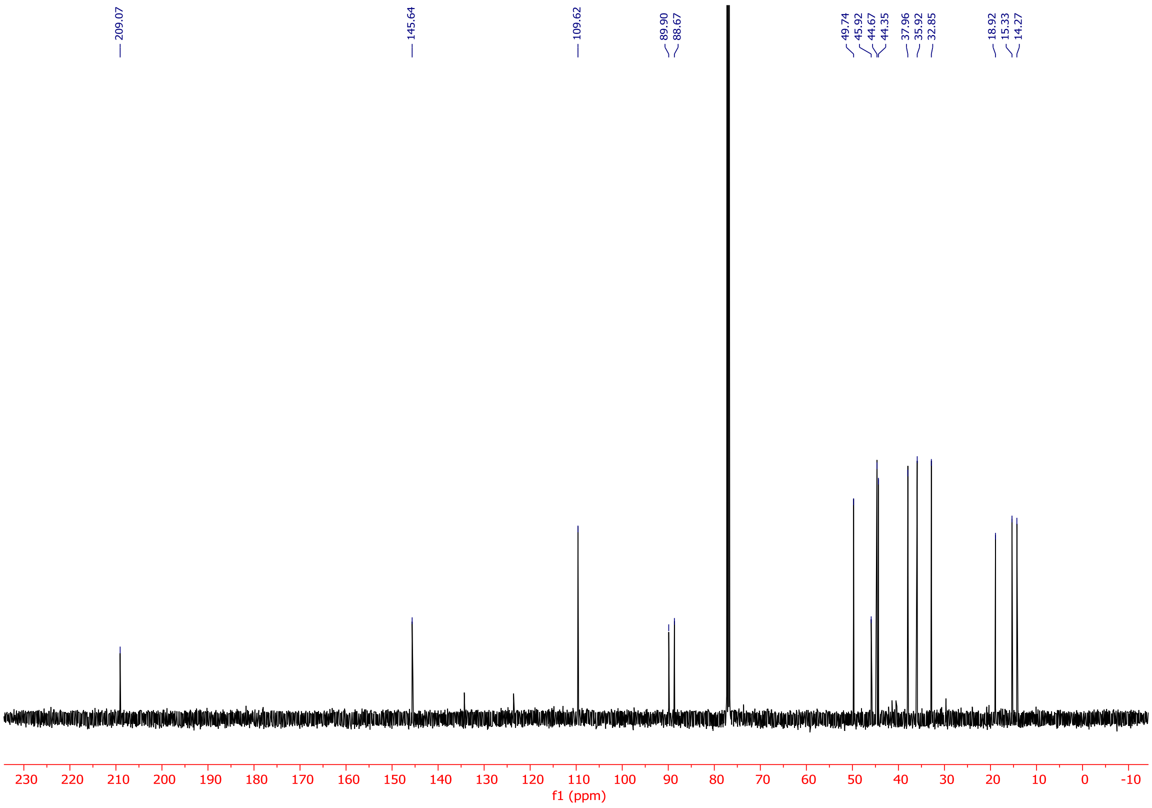
209.07
145.64
109.62
89.90
88.67
49.74
45.92
44.67
44.35
37.96
35.92
32.85
18.92
15.33
14.27
230 220 210 200 190 180 170 160 150 140 130 120 110 100 90 80 70 60 50 40 30 20 10 0 -10
f1 (ppm)

TOCSY

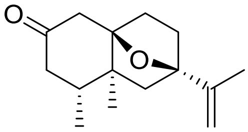

7

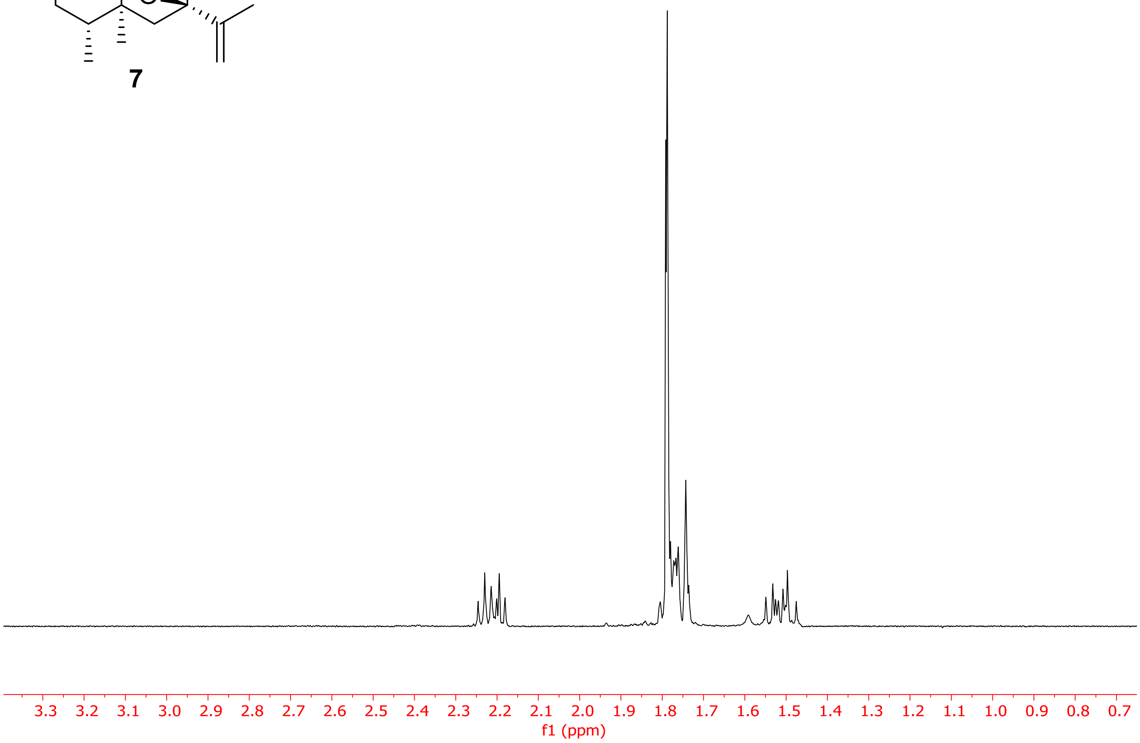

NOESY

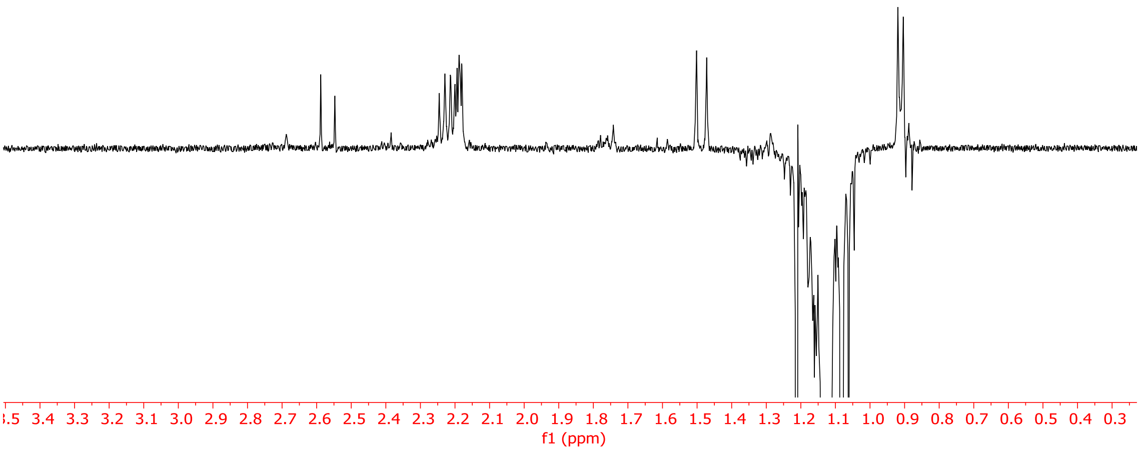

NOESY

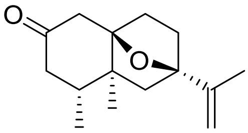

7

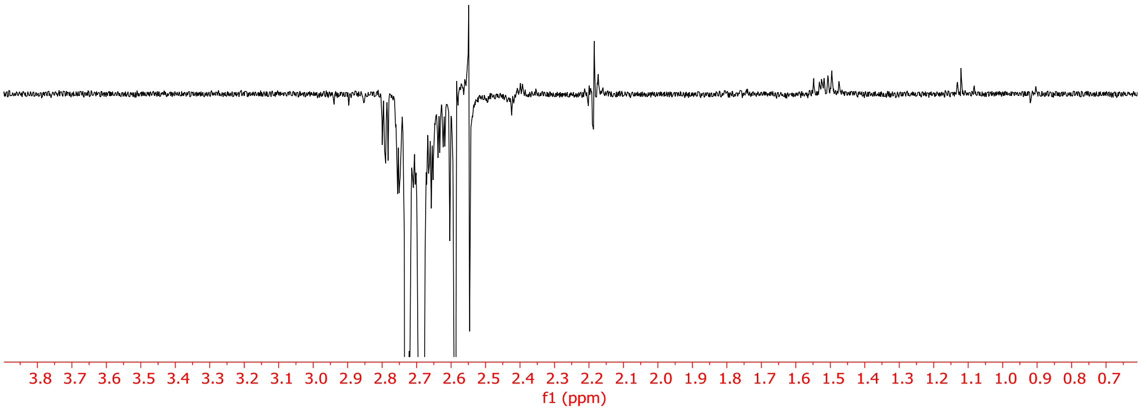

NOESY

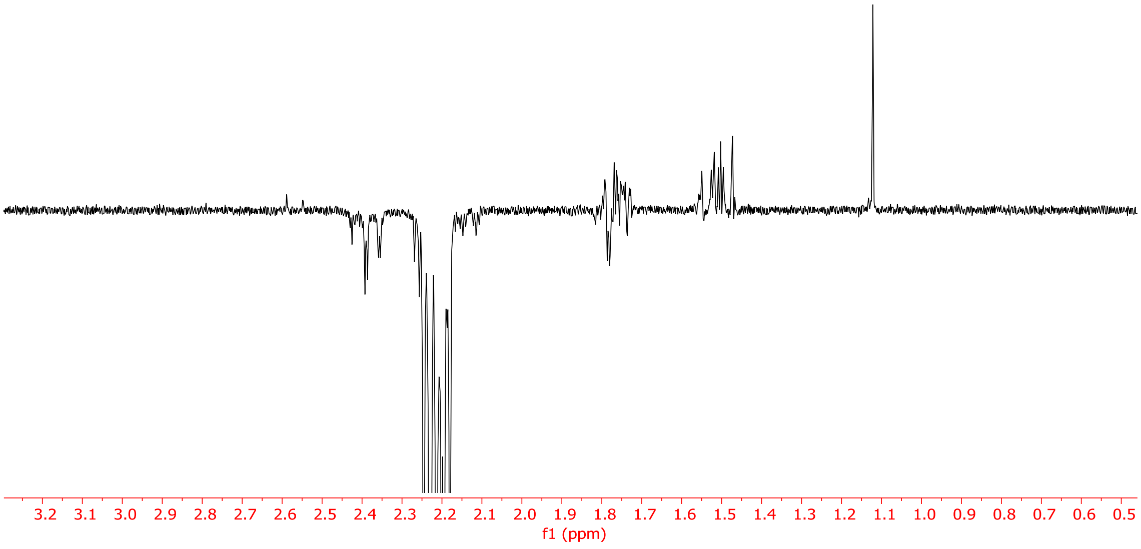

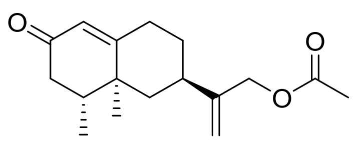

8

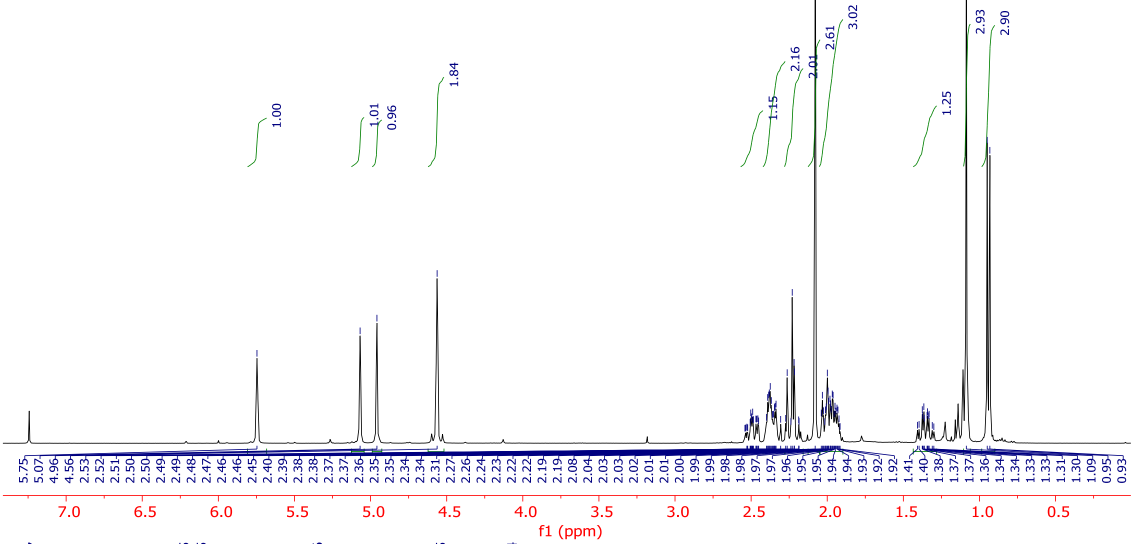
1.00
1.01
0.96
1.84
1.15
2.16
2.01
2.61
3.02
1.25
2.93
2.90
5.75 5.07 4.96 4.56 2.53 2.52 2.51 2.50 2.49 2.49 2.48 2.47 2.46 2.46 2.45 2.40 2.39 2.38 2.38 2.37 2.37 2.36 2.35 2.35 2.34 2.34 2.31 2.27 2.26 2.24 2.23 2.22 2.22 2.19 2.08 2.04 2.03 2.03 2.02 2.01 2.01 2.00 1.99 1.99 1.98 1.98 1.97 1.97 1.97 1.96 1.95 1.95 1.94 1.94 1.93 1.92 1.92 1.41 1.40 1.38 1.37 1.37 1.36 1.34 1.33 1.33 1.31 1.30 1.09 0.95 0.93
7.0 6.5 6.0 5.5 5.0 4.5 4.0 3.5 3.0 2.5 2.0 1.5 1.0 0.5
f1 (ppm)

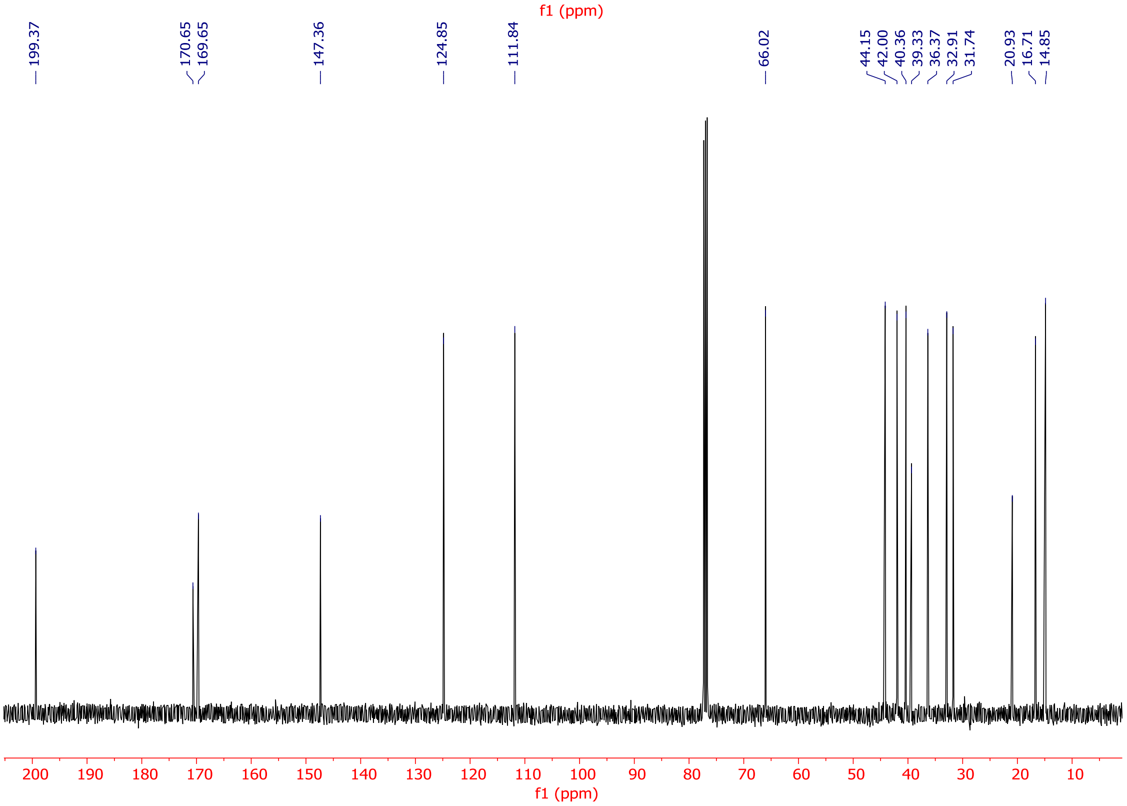
199.37
170.65
169.65
147.36
124.85
111.84
66.02
44.15
42.00
40.36
39.33
36.37
32.91
31.74
20.93
16.71
14.85
200 190 180 170 160 150 140 130 120 110 100 90 80 70 60 50 40 30 20 10
f1 (ppm)

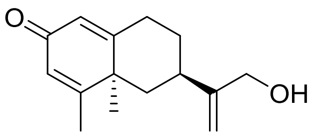
O
OH

9

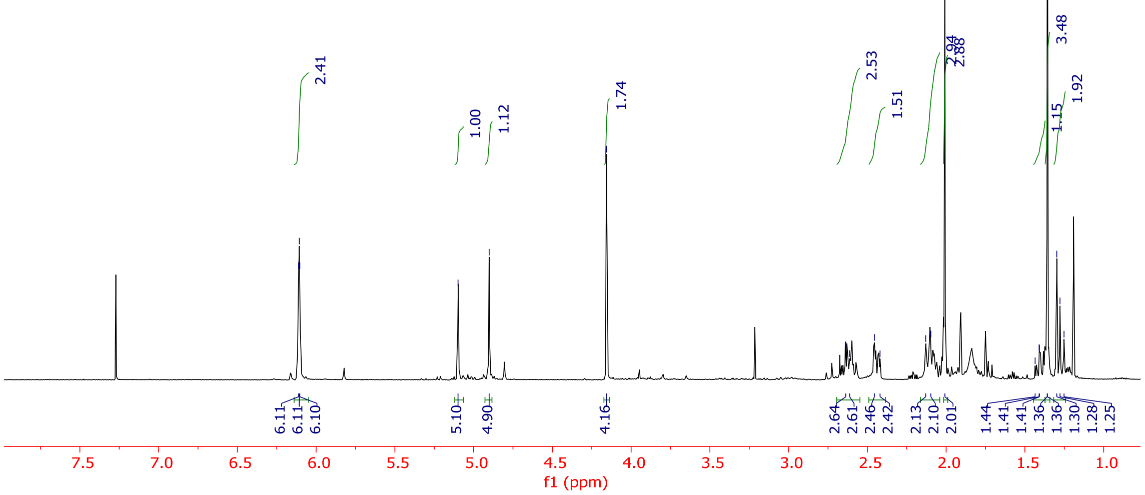
2.41
1.00
1.12
1.74
2.53
1.51
2.94
2.88
3.48
1.15
1.92
6.11
6.11
6.10
5.10
4.90
4.16
2.64
2.61
2.46
2.42
2.13
2.10
2.01
1.44
1.41
1.41
1.36
1.36
1.30
1.28
1.25
7.5
7.0
6.5
6.0
5.5
5.0
4.5
4.0
3.5
3.0
2.5
2.0
1.5
1.0
f1 (ppm)

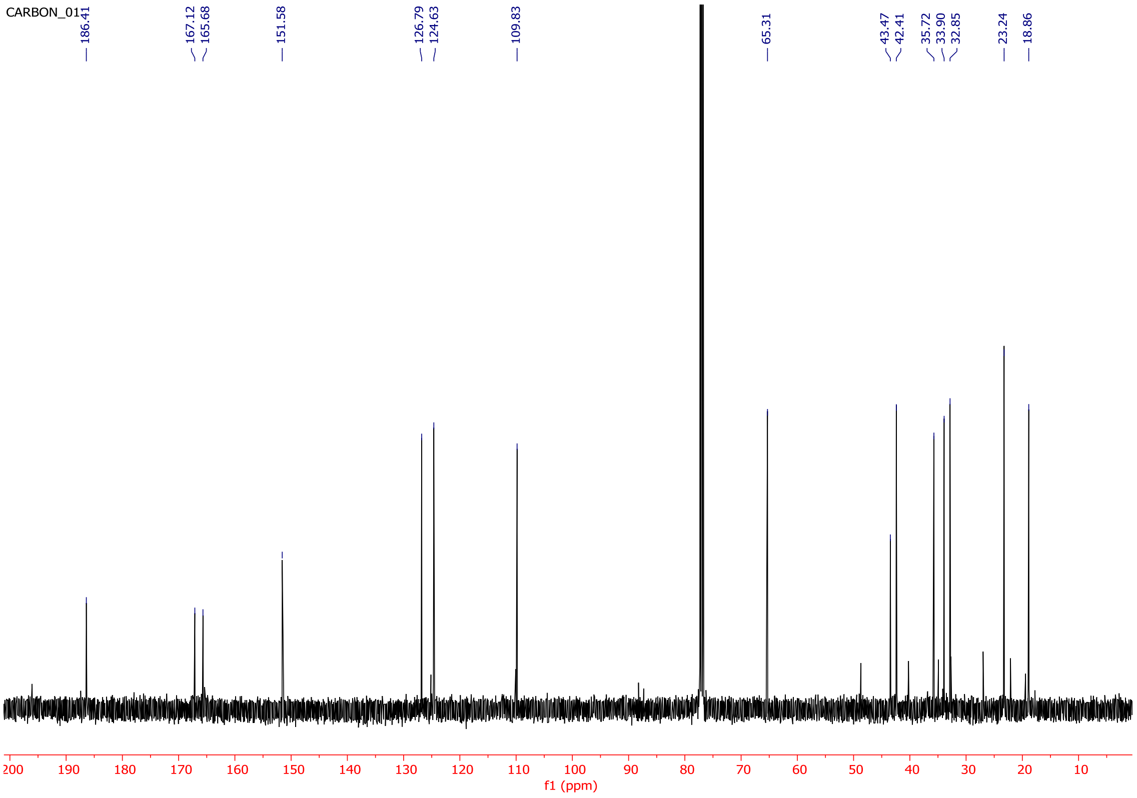
CARBON_011
186.41
167.12
165.68
151.58
126.79
124.63
109.83
65.31
43.47
42.41
35.72
33.90
32.85
23.24
18.86
200
190
180
170
160
150
140
130
120
110
100
90
80
70
60
50
40
30
20
10
f1 (ppm)

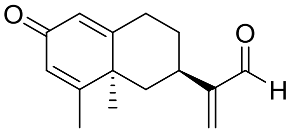

10

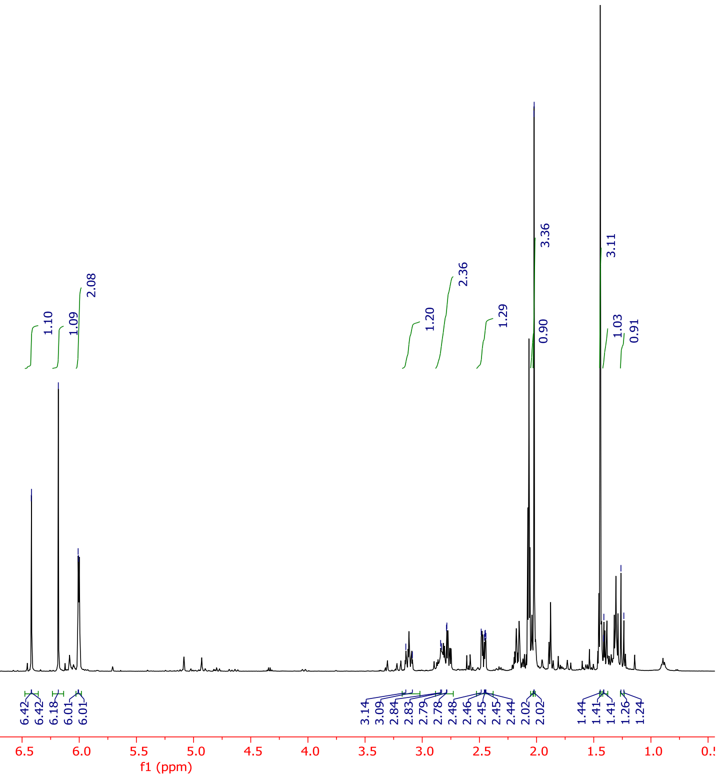

1.00
1.10
1.09
2.08
1.20
2.36
1.29
3.36
0.90
3.11
1.03
0.91
9.59
6.42
6.42
6.18
6.01
6.01
3.14
3.09
2.84
2.83
2.79
2.78
2.48
2.46
2.45
2.45
2.44
2.02
2.02
1.44
1.41
1.41
1.26
1.24
f1 (ppm)

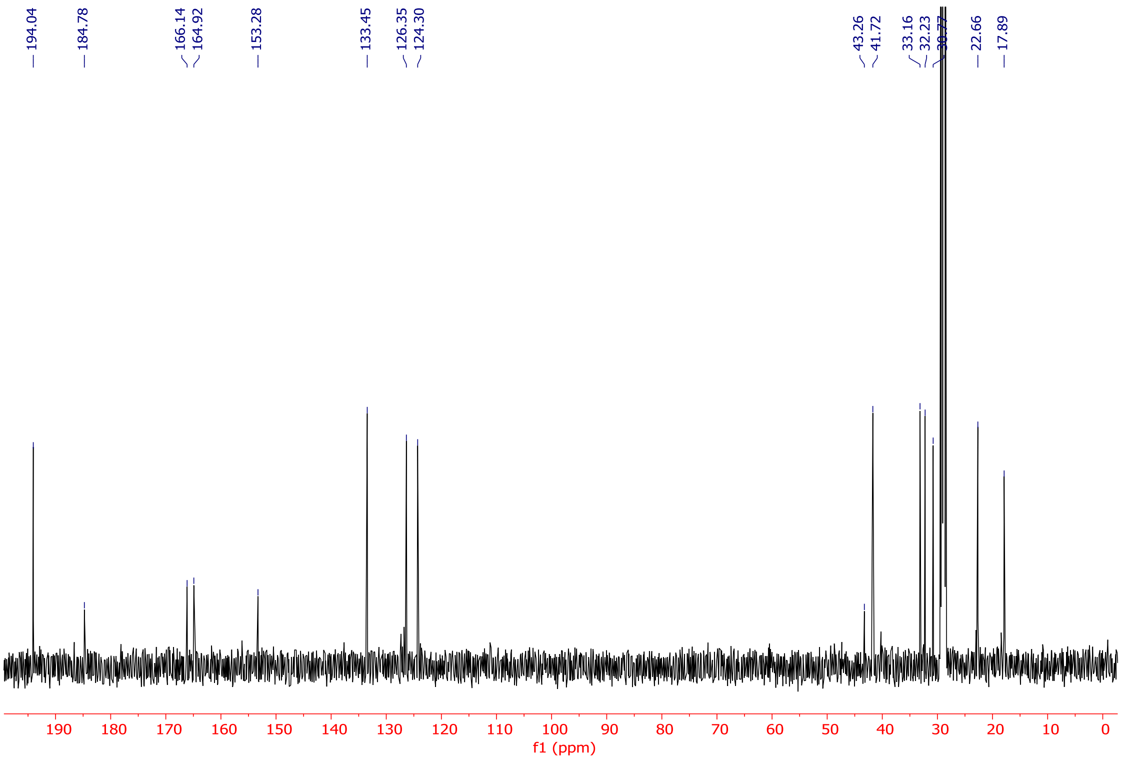

194.04
184.78
166.14
164.92
153.28
133.45
126.35
124.30
43.26
41.72
33.16
32.23
22.66
17.89
f1 (ppm)

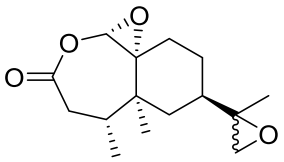
O
O
O
O

13

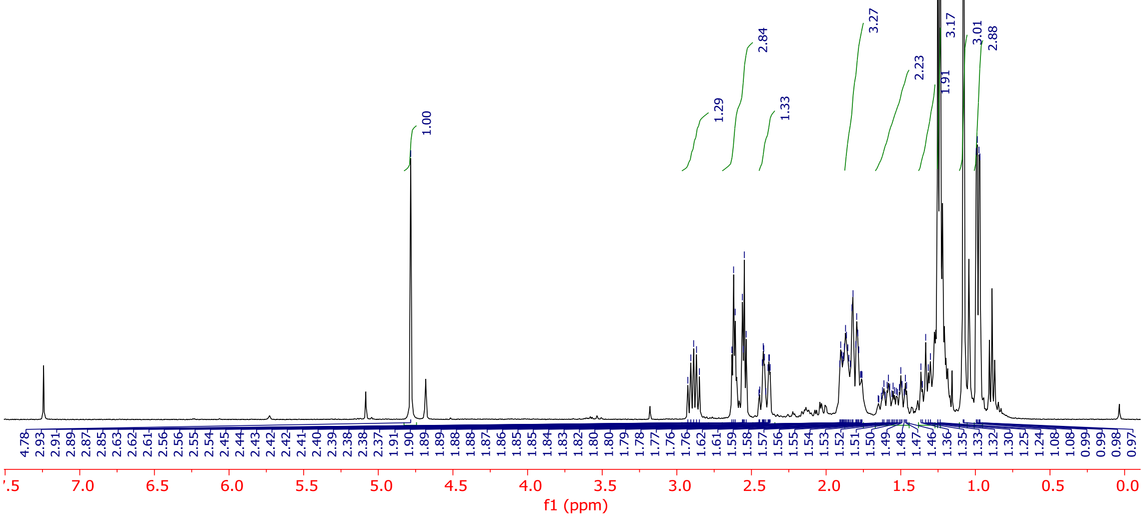
1.00
1.29
2.84
1.33
3.27
2.23
1.91
3.17
3.01
2.88
4.78 2.93 2.91 2.89 2.87 2.85 2.63 2.62 2.61 2.56 2.56 2.55 2.54 2.45 2.44 2.43 2.42 2.42 2.41 2.40 2.39 2.38 2.38 2.37 1.91 1.90 1.89 1.89 1.88 1.87 1.86 1.85 1.85 1.84 1.83 1.82 1.80 1.80 1.79 1.78 1.77 1.76 1.76 1.62 1.61 1.59 1.58 1.57 1.56 1.55 1.54 1.53 1.52 1.51 1.50 1.49 1.48 1.47 1.46 1.36 1.35 1.33 1.32 1.30 1.25 1.24 1.08 1.08 0.99 0.99 0.98 0.97
7.0 6.5 6.0 5.5 5.0 4.5 4.0 3.5 3.0 2.5 2.0 1.5 1.0 0.5 0.0
f1 (ppm)

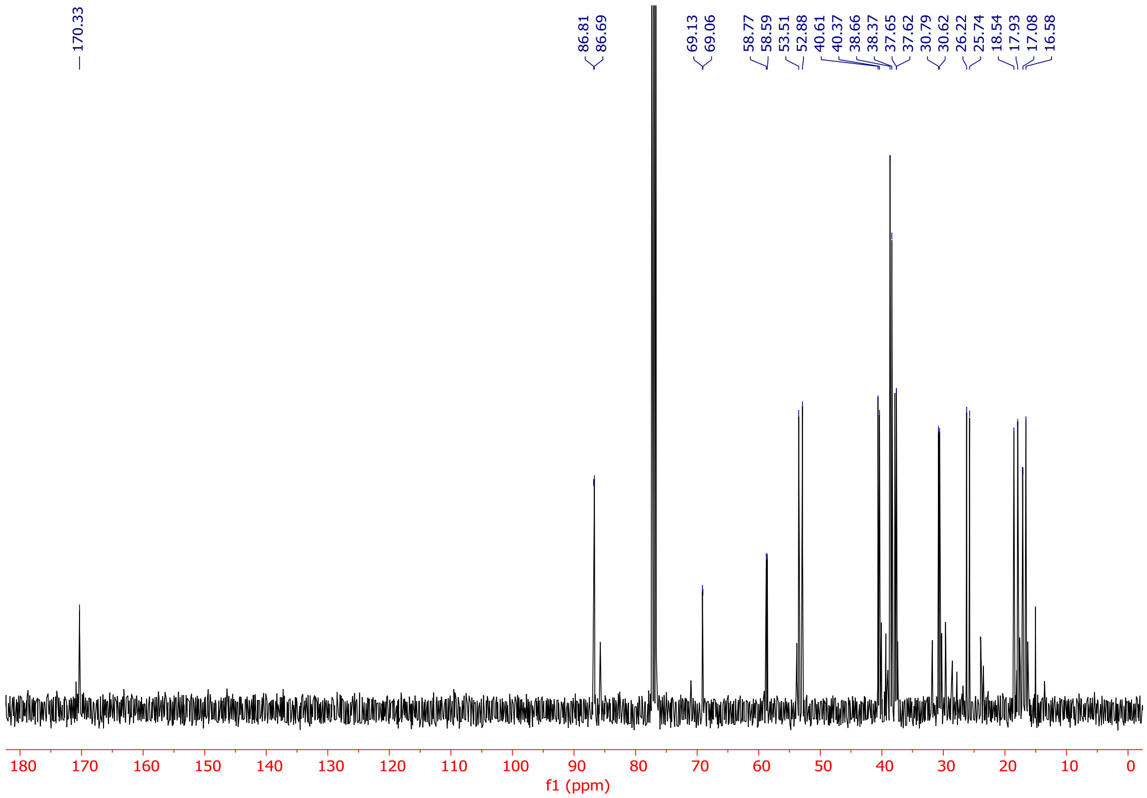
170.33
86.81
86.69
69.13
69.06
58.77
58.59
53.51
52.88
40.61
40.37
38.66
38.37
37.65
37.62
30.79
30.62
26.22
25.74
18.54
17.93
17.08
16.58
180 170 160 150 140 130 120 110 100 90 80 70 60 50 40 30 20 10 0
f1 (ppm)

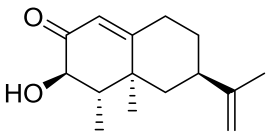
O
HO

14

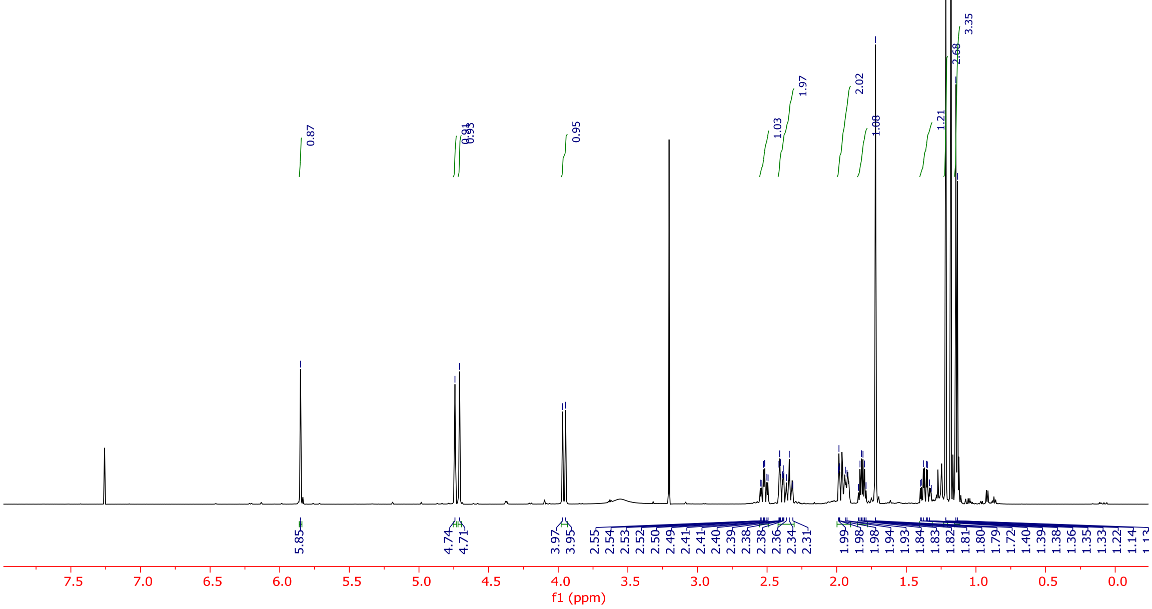
0.87
0.95
1.03
1.97
2.02
1.00
1.21
2.68
3.35
5.85
4.74
4.71
3.97
3.95
2.55
2.54
2.53
2.52
2.50
2.49
2.41
2.41
2.40
2.39
2.38
2.38
2.36
2.34
2.31
1.99
1.98
1.98
1.94
1.93
1.84
1.83
1.82
1.81
1.80
1.79
1.72
1.40
1.39
1.38
1.36
1.35
1.33
1.22
1.14
7.5
7.0
6.5
6.0
5.5
5.0
4.5
4.0
3.5
3.0
2.5
2.0
1.5
1.0
0.5
0.0
f1 (ppm)

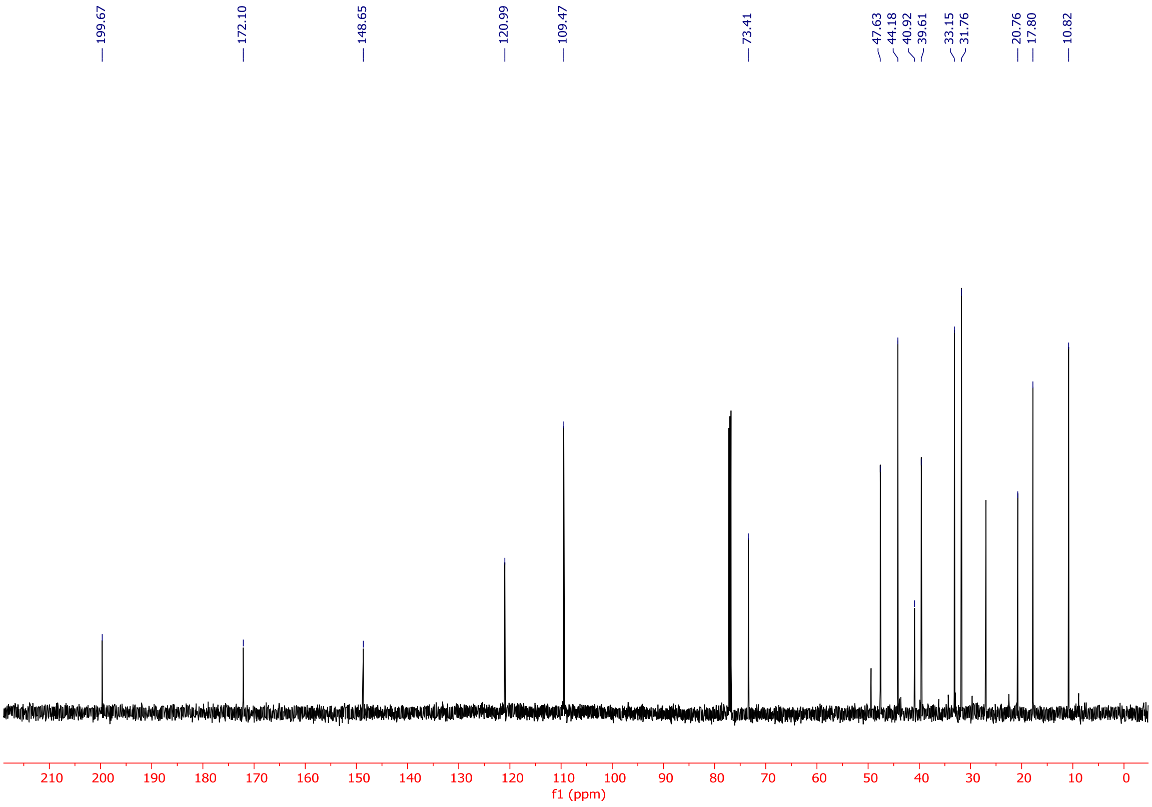
199.67
172.10
148.65
120.99
109.47
73.41
47.63
44.18
40.92
39.61
33.15
31.76
20.76
17.80
10.82
210
200
190
180
170
160
150
140
130
120
110
100
90
80
70
60
50
40
30
20
10
0
f1 (ppm)

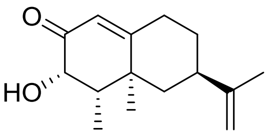
O
HO

15

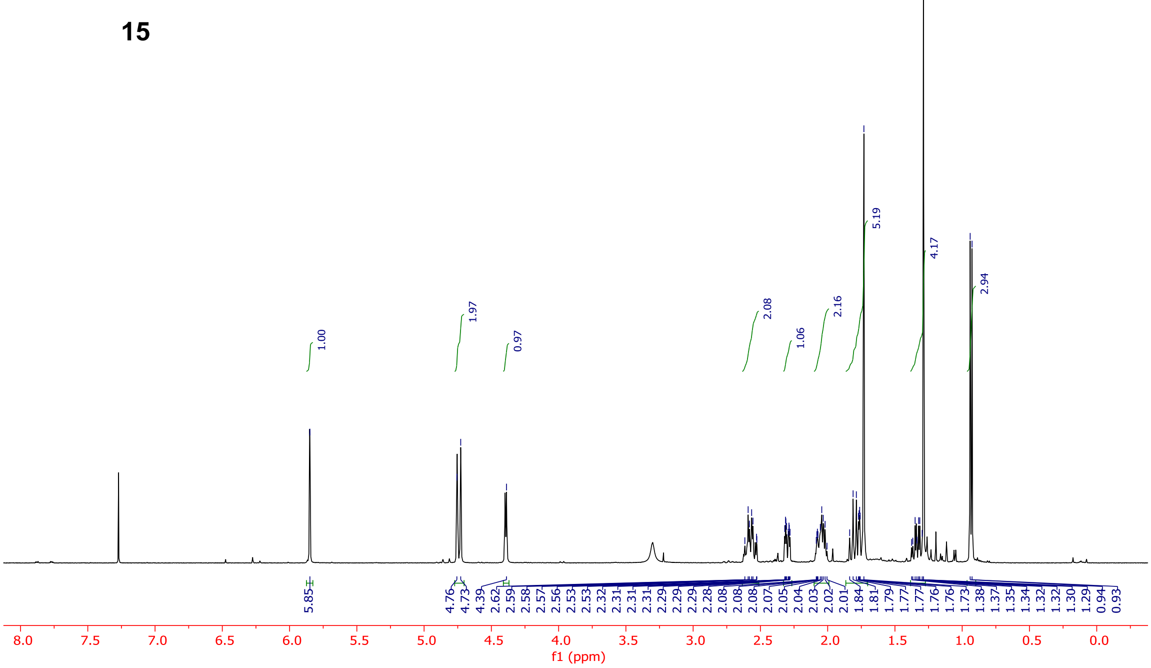
1.00
1.97
0.97
2.08
1.06
2.16
5.19
4.17
2.94
5.85
4.76
4.73
4.39
2.62
2.59
2.58
2.57
2.56
2.53
2.53
2.32
2.31
2.31
2.31
2.29
2.29
2.29
2.28
2.08
2.08
2.07
2.05
2.04
2.03
2.02
2.01
1.84
1.81
1.79
1.77
1.77
1.76
1.76
1.73
1.38
1.37
1.35
1.34
1.32
1.32
1.30
1.29
0.94
0.93
8.0 7.5 7.0 6.5 6.0 5.5 5.0 4.5 4.0 3.5 3.0 2.5 2.0 1.5 1.0 0.5 0.0
f1 (ppm)

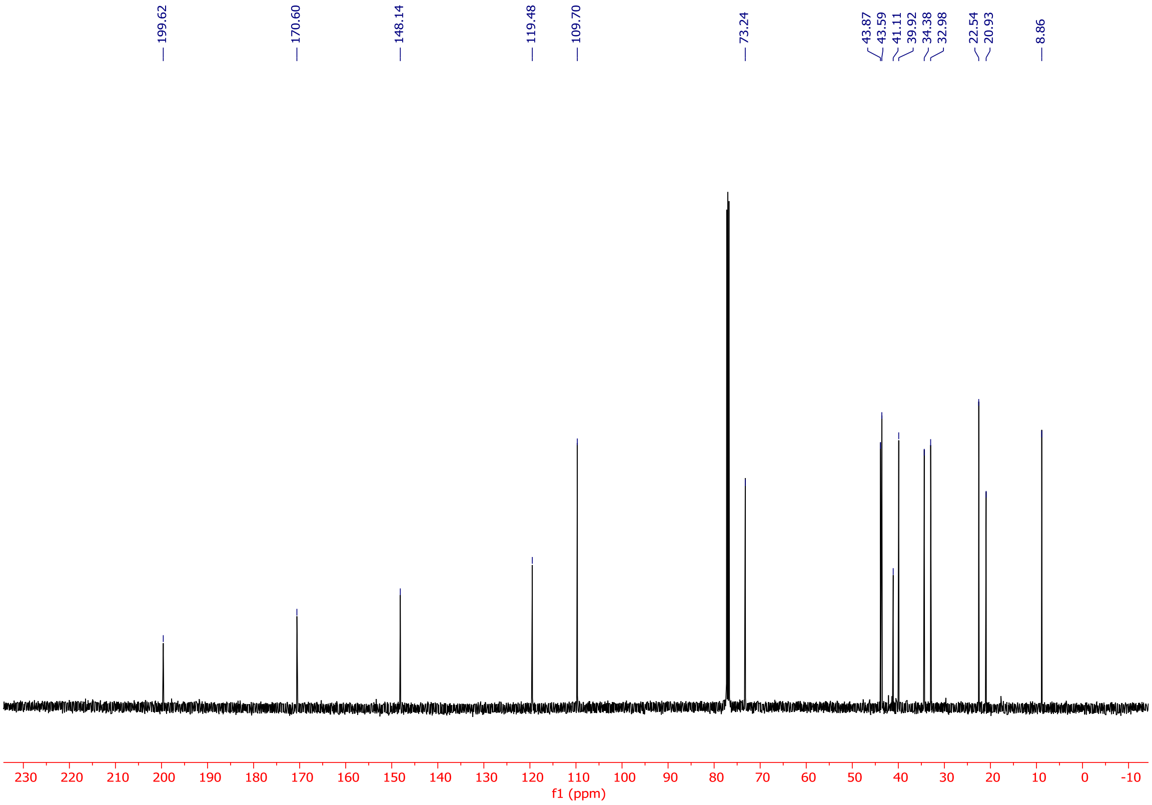
199.62
170.60
148.14
119.48
109.70
73.24
43.87
43.59
41.11
39.92
34.38
32.98
22.54
20.93
8.86
230 220 210 200 190 180 170 160 150 140 130 120 110 100 90 80 70 60 50 40 30 20 10 0 -10
f1 (ppm)

NOEDIFF

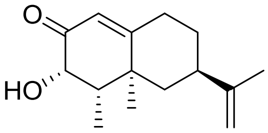

15

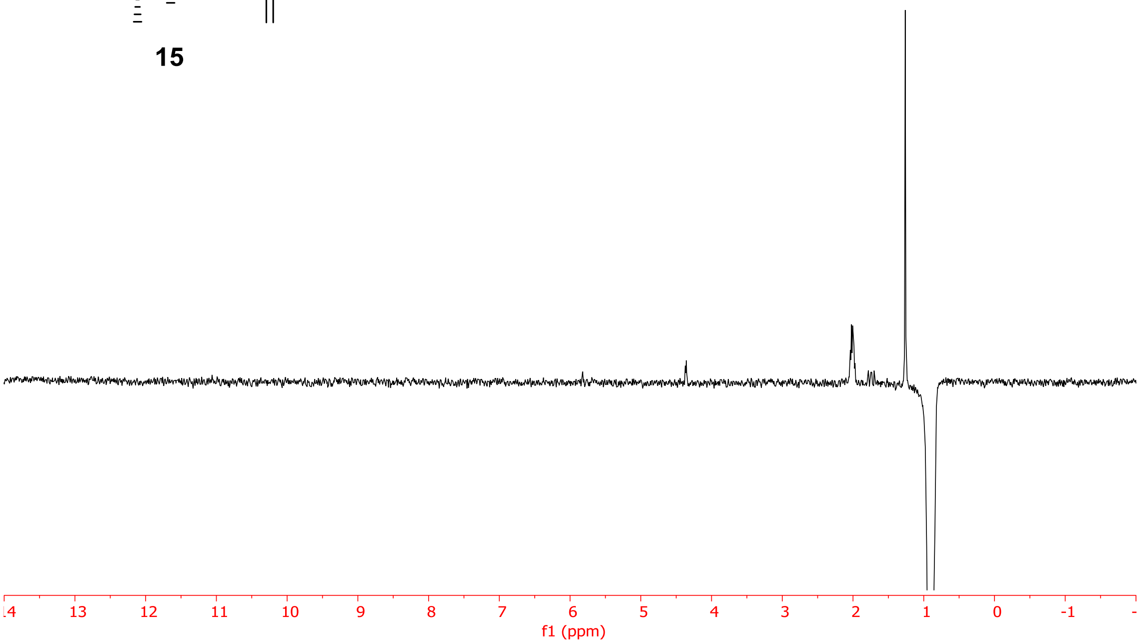

NOEDIFF

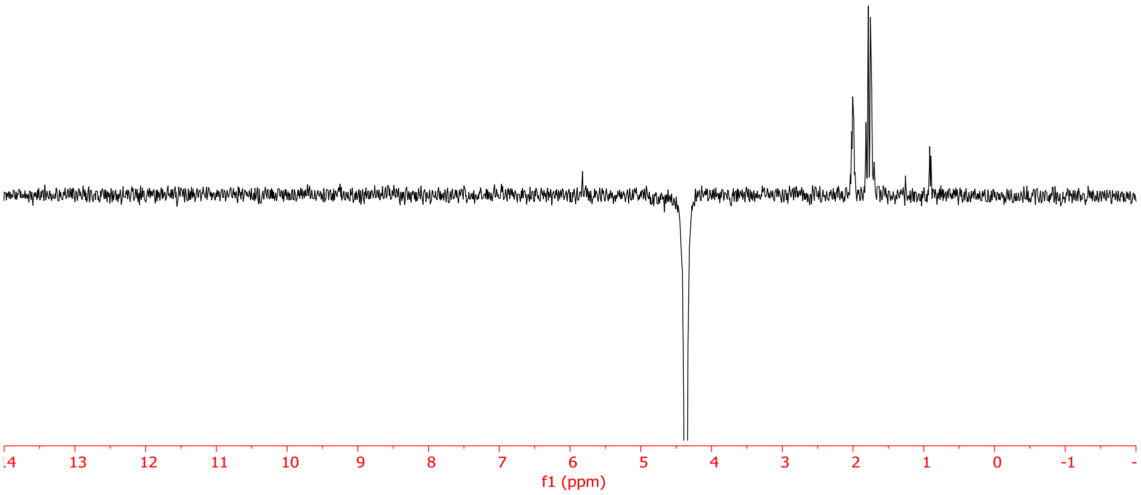

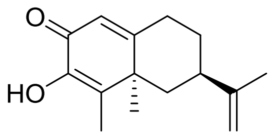
O
HO

16

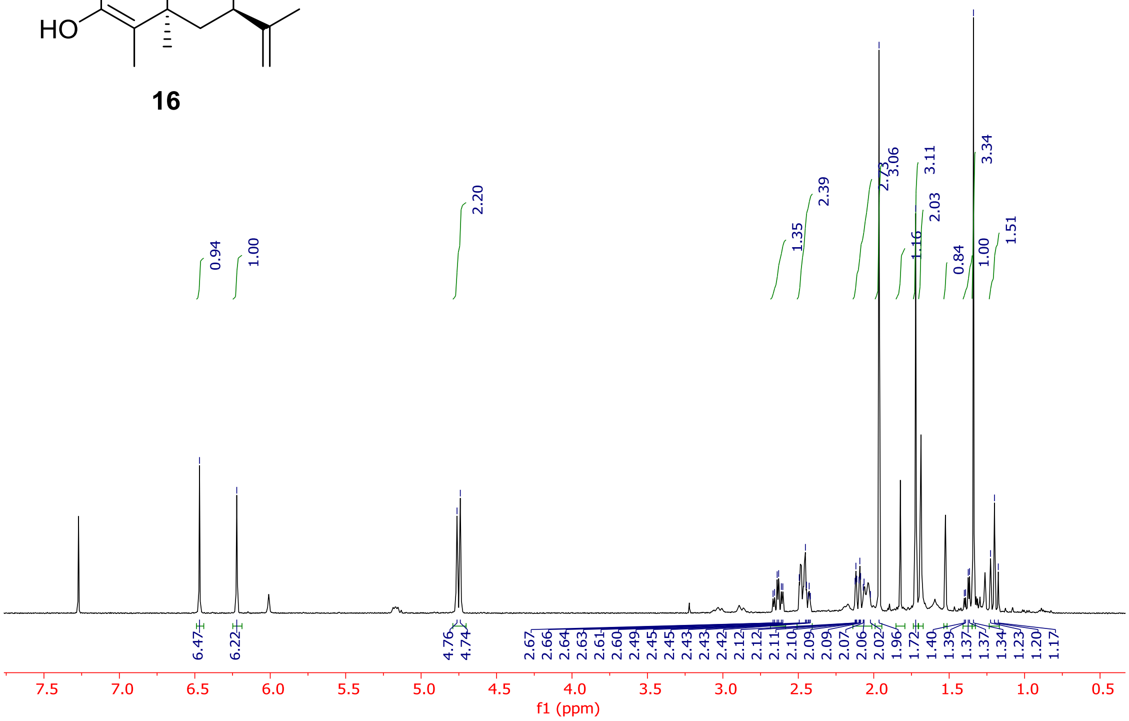
0.94
1.00
2.20
1.35
2.39
2.73
3.06
1.16
3.11
2.03
0.84
1.00
3.34
1.51
6.47
6.22
4.76
4.74
2.67
2.66
2.64
2.63
2.61
2.60
2.49
2.45
2.45
2.43
2.43
2.42
2.12
2.12
2.11
2.10
2.09
2.09
2.07
2.06
2.02
1.96
1.72
1.40
1.39
1.37
1.37
1.34
1.23
1.20
1.17
7.5
7.0
6.5
6.0
5.5
5.0
4.5
4.0
3.5
3.0
2.5
2.0
1.5
1.0
0.5
f1 (ppm)

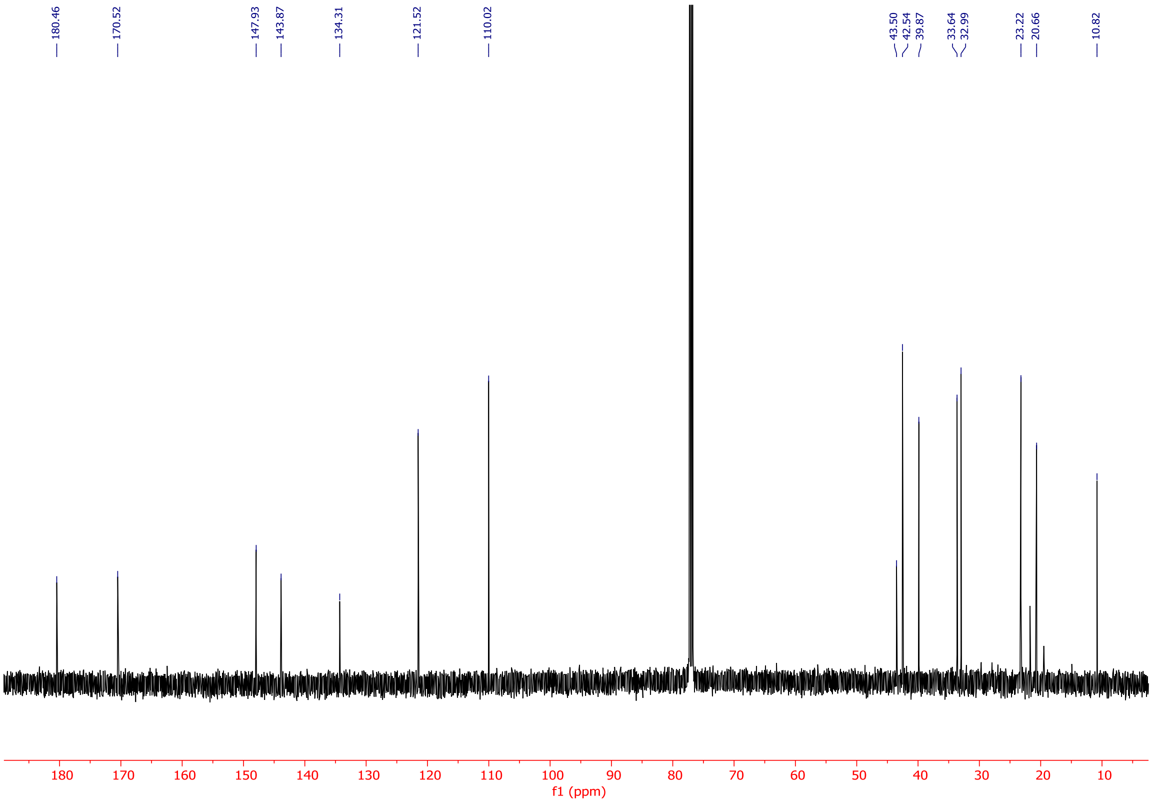
180.46
170.52
147.93
143.87
134.31
121.52
110.02
43.50
42.54
39.87
33.64
32.99
23.22
20.66
10.82
180
170
160
150
140
130
120
110
100
90
80
70
60
50
40
30
20
10
f1 (ppm)

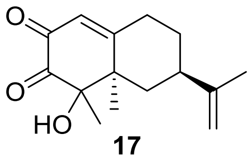
O
O
HO

17

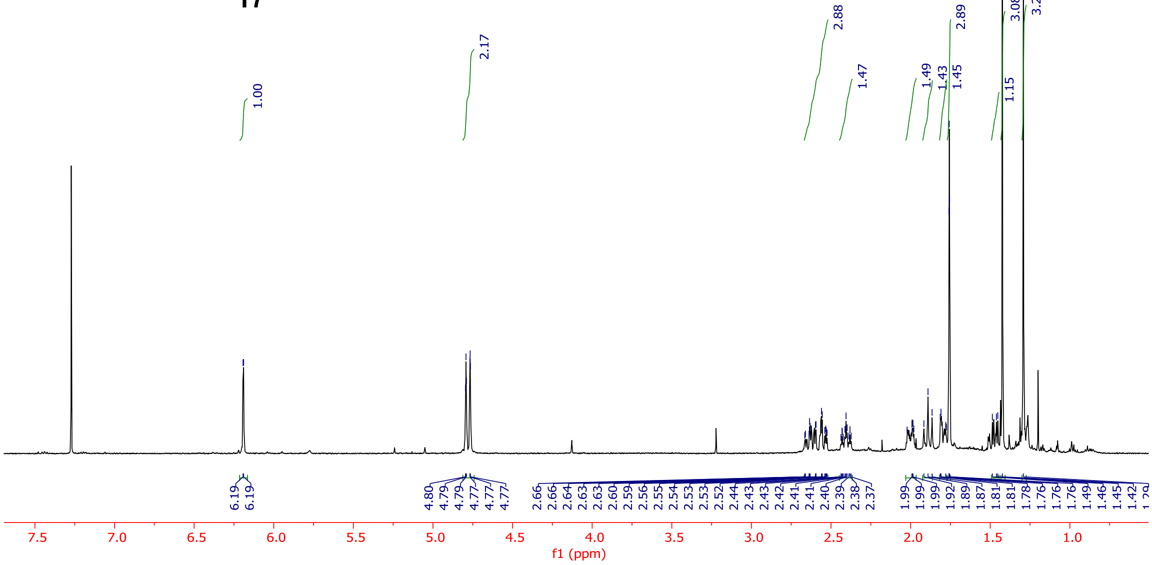
1.00
2.17
2.88
1.47
1.49
1.43
1.45
2.89
1.15
3.08
3.23
6.19
6.19
4.80
4.79
4.79
4.77
4.77
4.77
2.66
2.66
2.64
2.63
2.63
2.60
2.59
2.56
2.55
2.54
2.53
2.53
2.52
2.44
2.43
2.43
2.42
2.41
2.41
2.40
2.39
2.38
2.37
1.99
1.99
1.99
1.92
1.89
1.87
1.81
1.81
1.78
1.76
1.76
1.76
1.49
1.46
1.45
1.42
7.5
7.0
6.5
6.0
5.5
5.0
4.5
4.0
3.5
3.0
2.5
2.0
1.5
1.0
f1 (ppm)

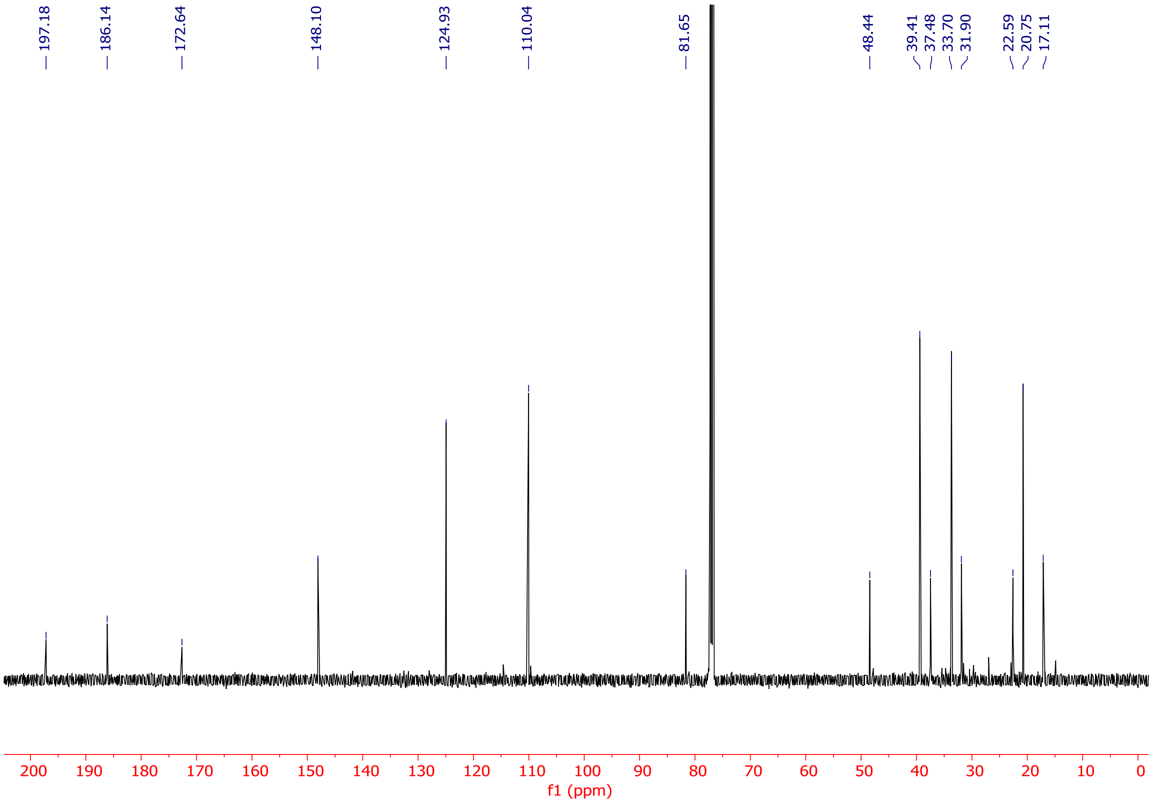
197.18
186.14
172.64
148.10
124.93
110.04
81.65
48.44
39.41
37.48
33.70
31.90
22.59
20.75
17.11
200
190
180
170
160
150
140
130
120
110
100
90
80
70
60
50
40
30
20
10
0
f1 (ppm)

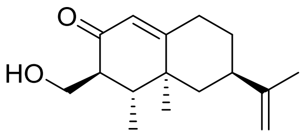
O
HO

18

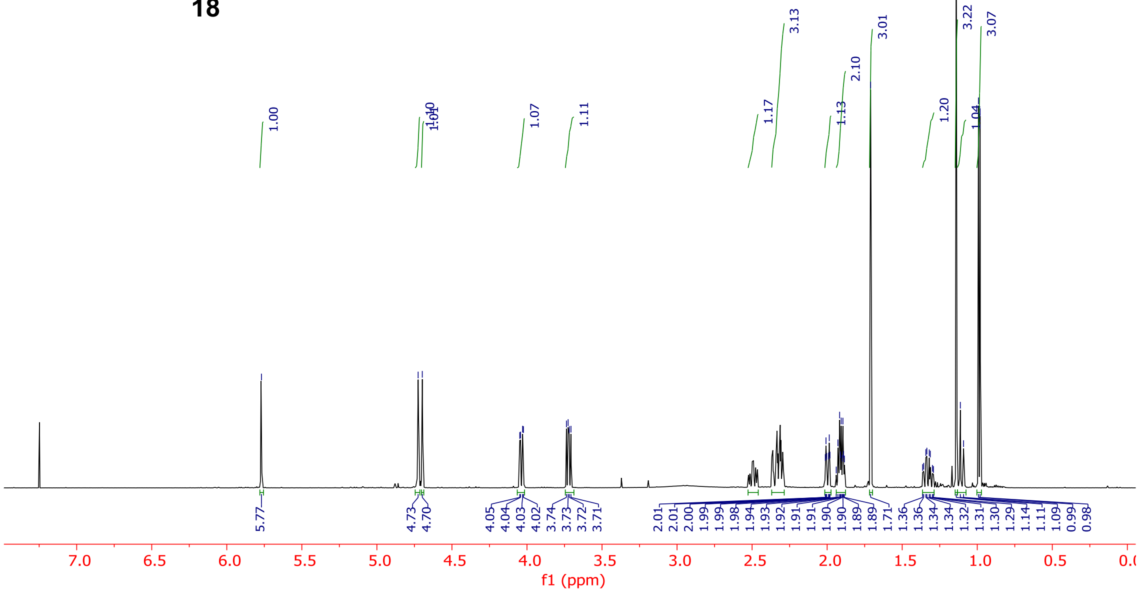
1.00
1.00
1.01
1.07
1.11
1.17
3.13
1.13
2.10
3.01
1.20
3.22
1.04
3.07
5.77
4.73
4.70
4.05
4.04
4.03
4.02
3.74
3.73
3.72
3.71
2.01
2.01
2.00
1.99
1.99
1.98
1.94
1.93
1.92
1.91
1.91
1.90
1.90
1.89
1.89
1.71
1.36
1.36
1.34
1.34
1.32
1.31
1.30
1.29
1.14
1.11
1.09
0.99
0.98
7.0
6.5
6.0
5.5
5.0
4.5
4.0
3.5
3.0
2.5
2.0
1.5
1.0
0.5
f1 (ppm)

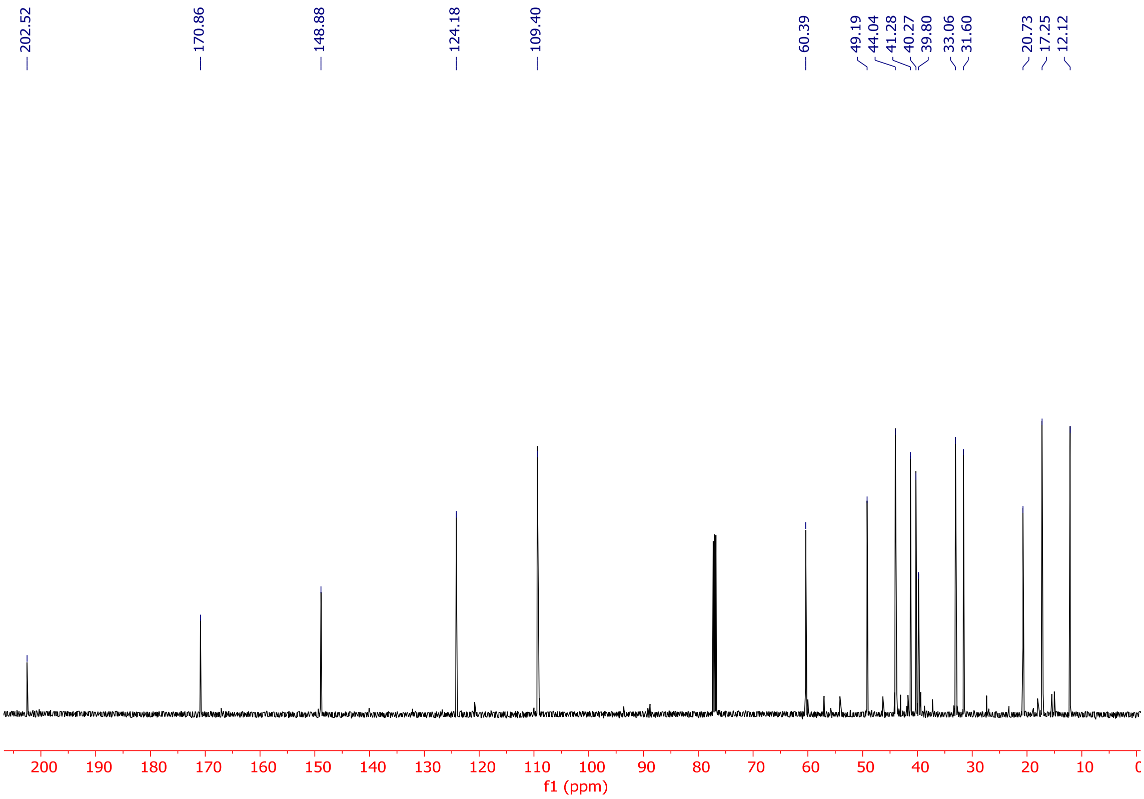
202.52
170.86
148.88
124.18
109.40
60.39
49.19
44.04
41.28
40.27
39.80
33.06
31.60
20.73
17.25
12.12
200
190
180
170
160
150
140
130
120
110
100
90
80
70
60
50
40
30
20
10
f1 (ppm)

TOCSY

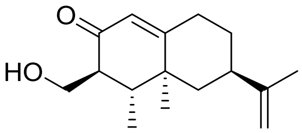

18

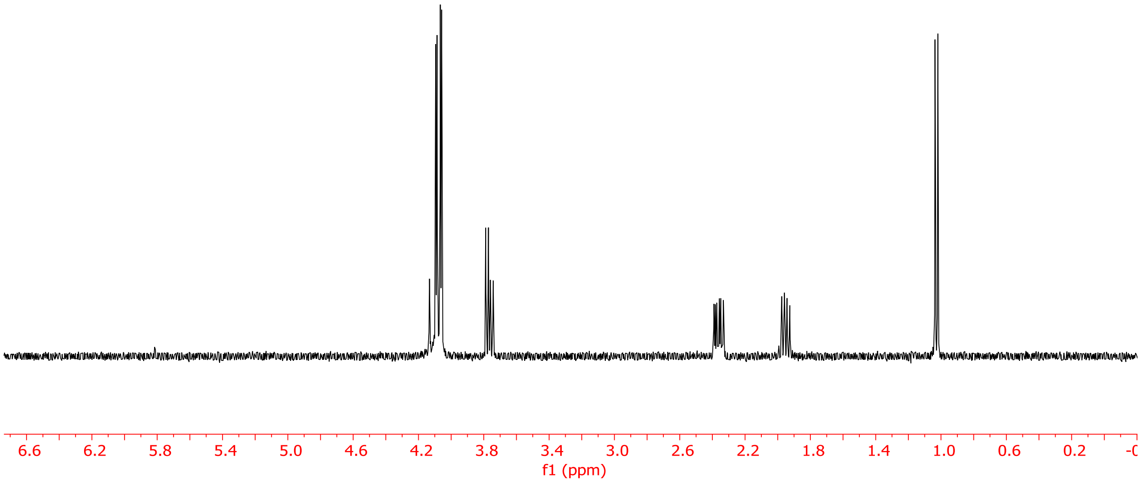

NOESY

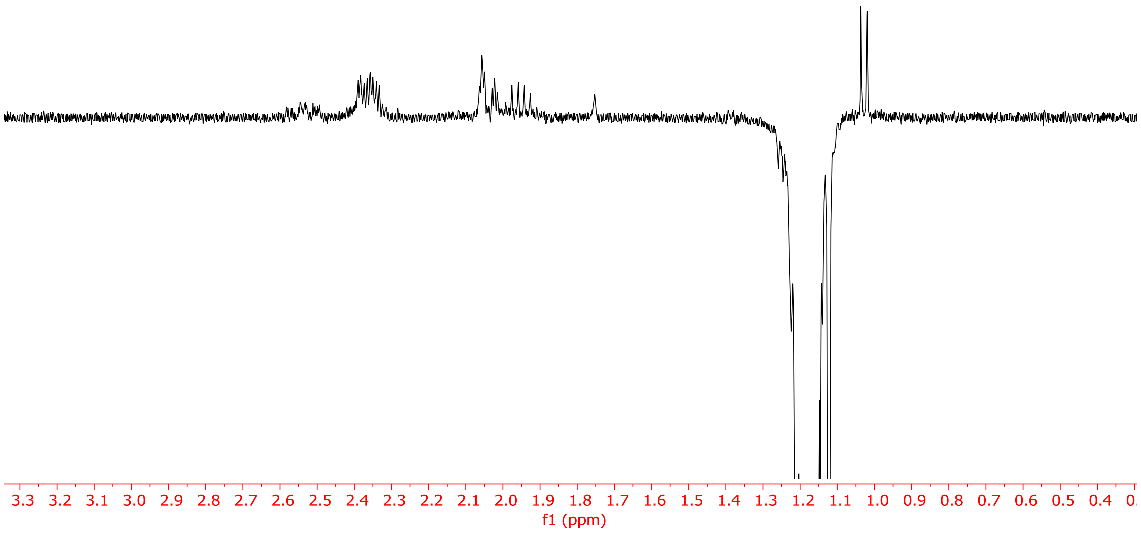

NOESY

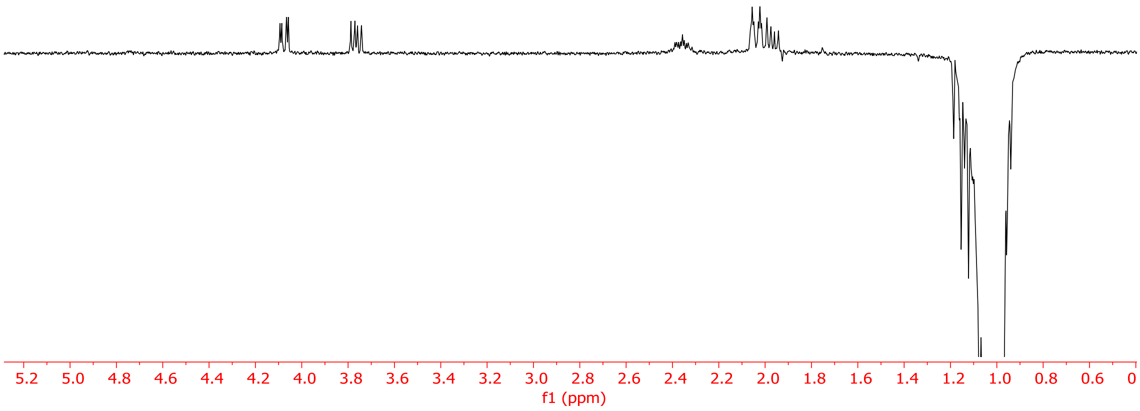
5.2 5.0 4.8 4.6 4.4 4.2 4.0 3.8 3.6 3.4 3.2 3.0 2.8 2.6 2.4 2.2 2.0 1.8 1.6 1.4 1.2 1.0 0.8 0.6 0
f1 (ppm)

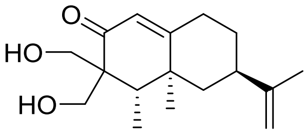
O
HO
HO

19

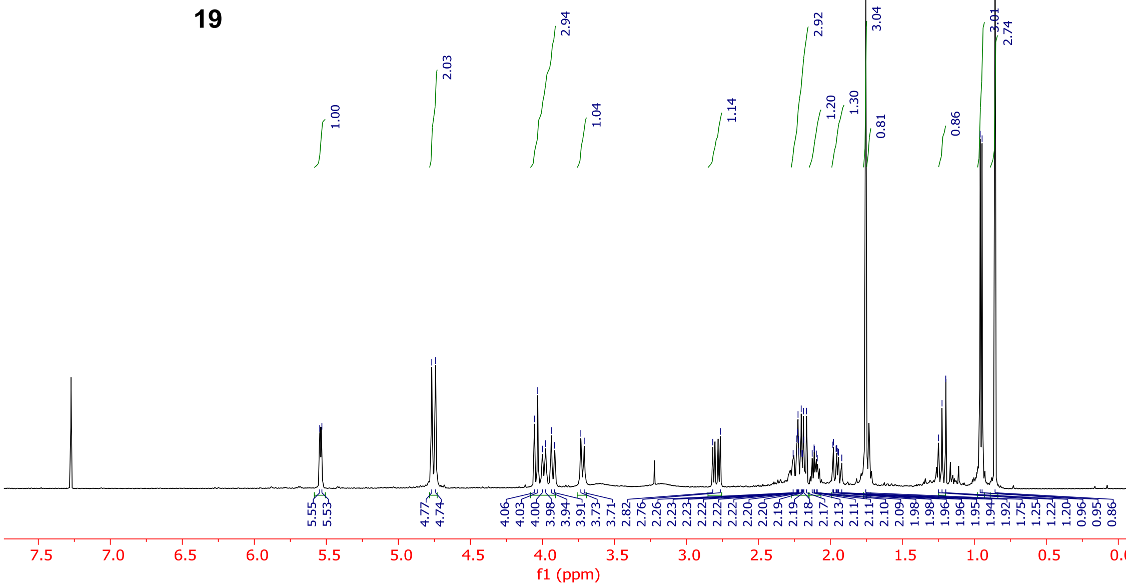
1.00
2.03
2.94
1.04
1.14
2.92
1.20
1.30
3.04
0.81
0.86
3.01
2.74
5.55
5.53
4.77
4.74
4.06
4.03
4.00
3.98
3.94
3.91
3.73
3.71
2.82
2.76
2.26
2.23
2.23
2.22
2.22
2.22
2.20
2.20
2.19
2.18
2.17
2.13
2.11
2.11
2.10
2.09
1.98
1.98
1.96
1.96
1.95
1.94
1.92
1.75
1.25
1.22
1.20
0.96
0.95
0.86
f1 (ppm)

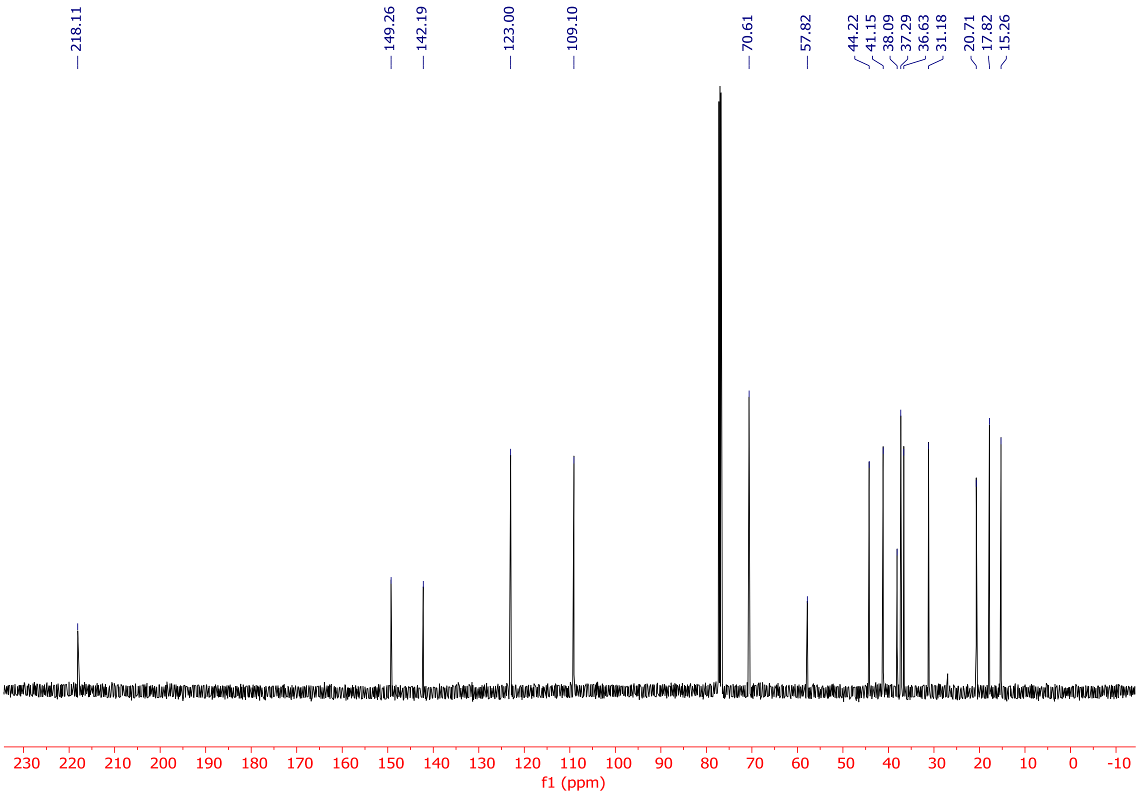
218.11
149.26
142.19
123.00
109.10
70.61
57.82
44.22
41.15
38.09
37.29
36.63
31.18
20.71
17.82
15.26
f1 (ppm)

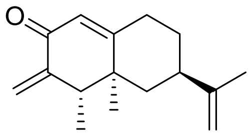
O

20

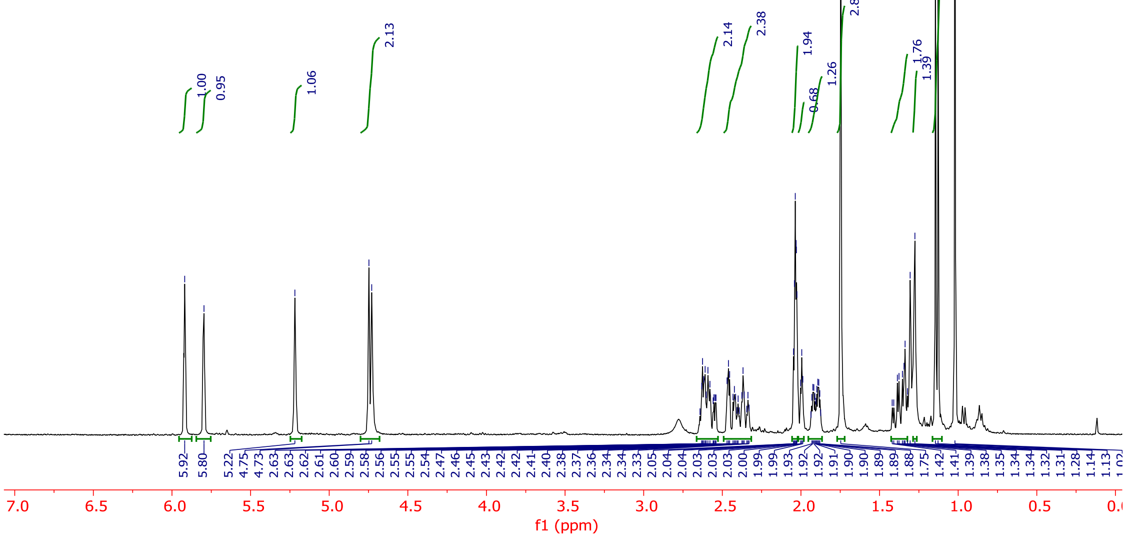
2.13
1.00
0.95
1.06
2.14
2.38
1.94
0.68
1.26
2.83
1.76
1.39
3.26
f1 (ppm)

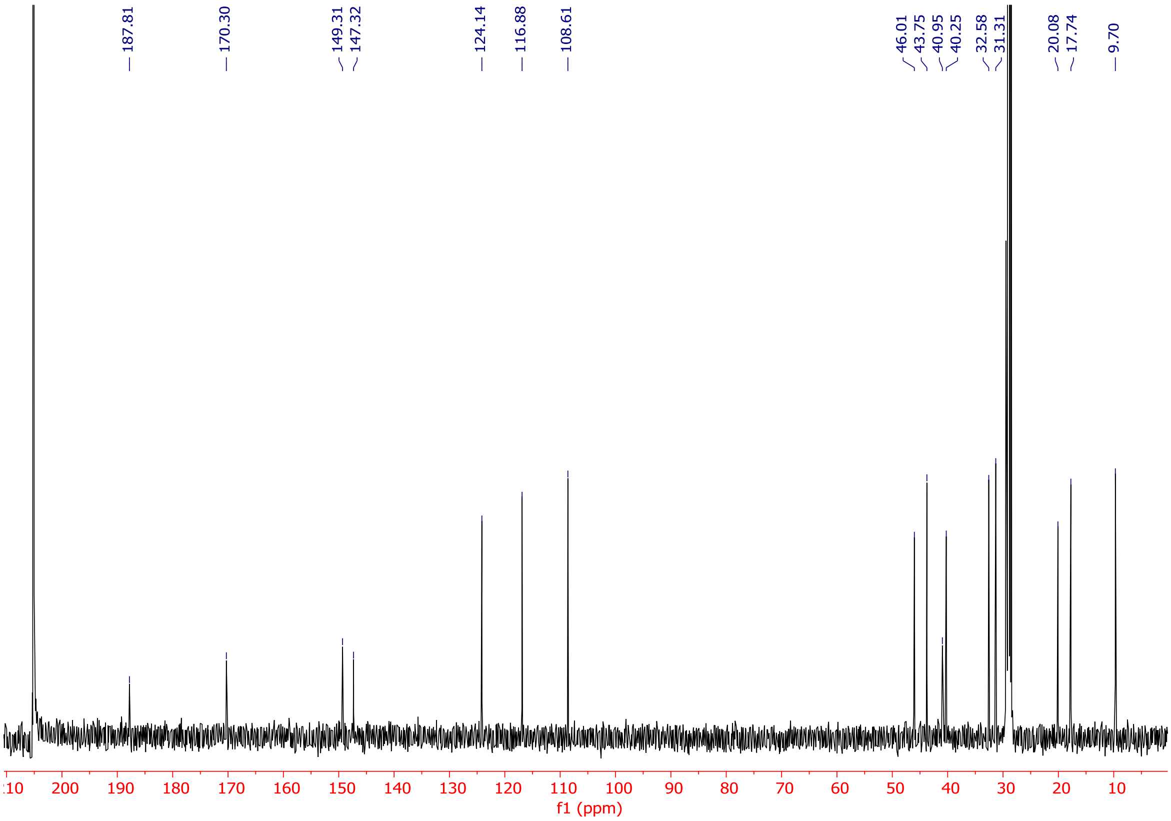
187.81
170.30
149.31
147.32
124.14
116.88
108.61
46.01
43.75
40.95
40.25
32.58
31.31
20.08
17.74
9.70
f1 (ppm)

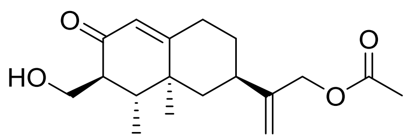

21

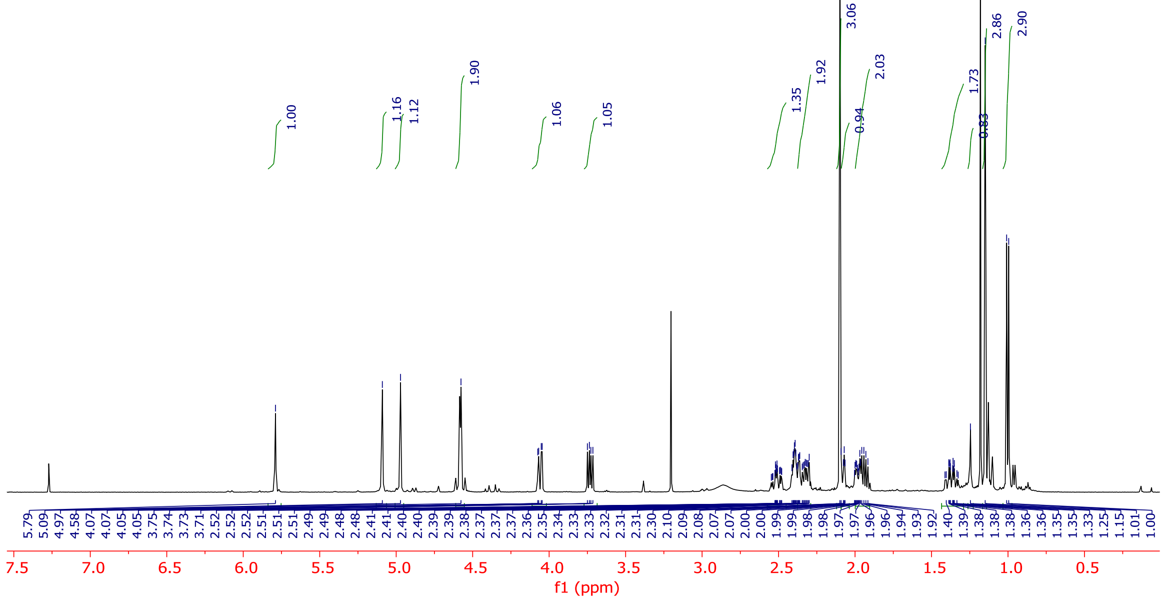
f1 (ppm)

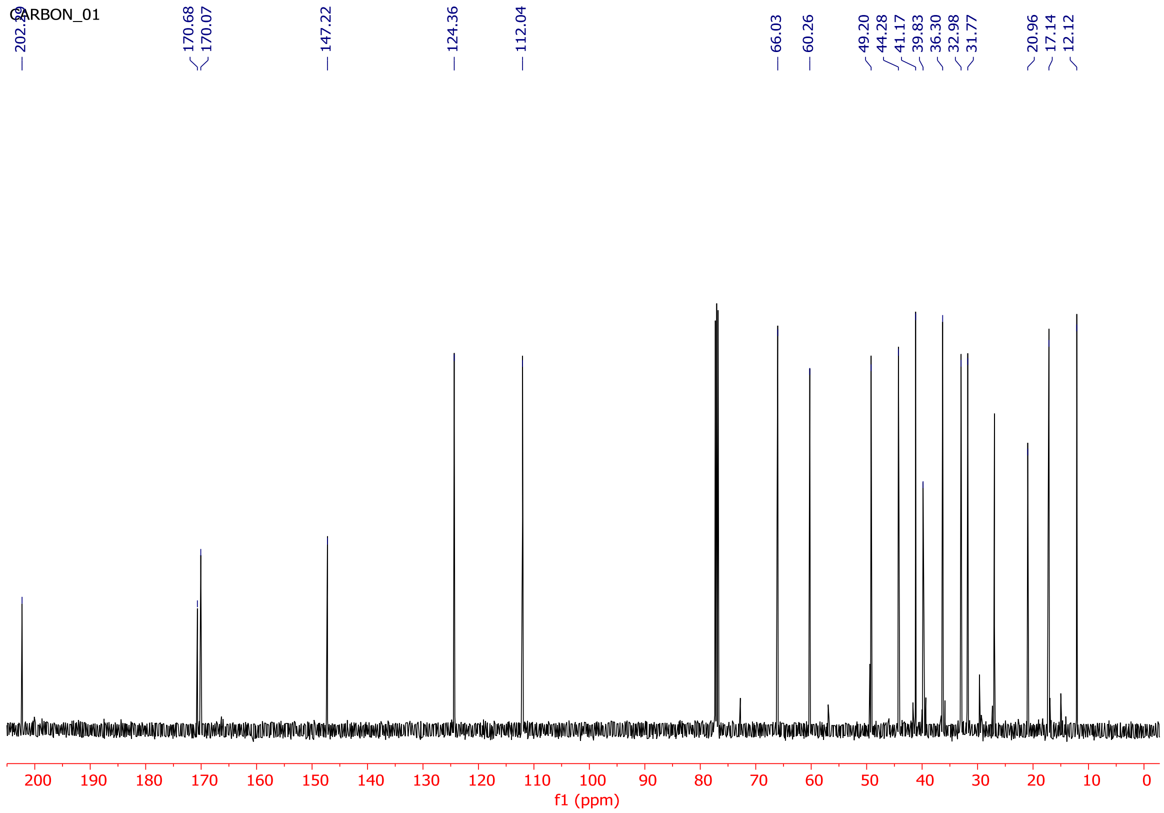
CARBON_01
202.39
170.68
170.07
147.22
124.36
112.04
66.03
60.26
49.20
44.28
41.17
39.83
36.30
32.98
31.77
20.96
17.14
12.12
f1 (ppm)

NOESY

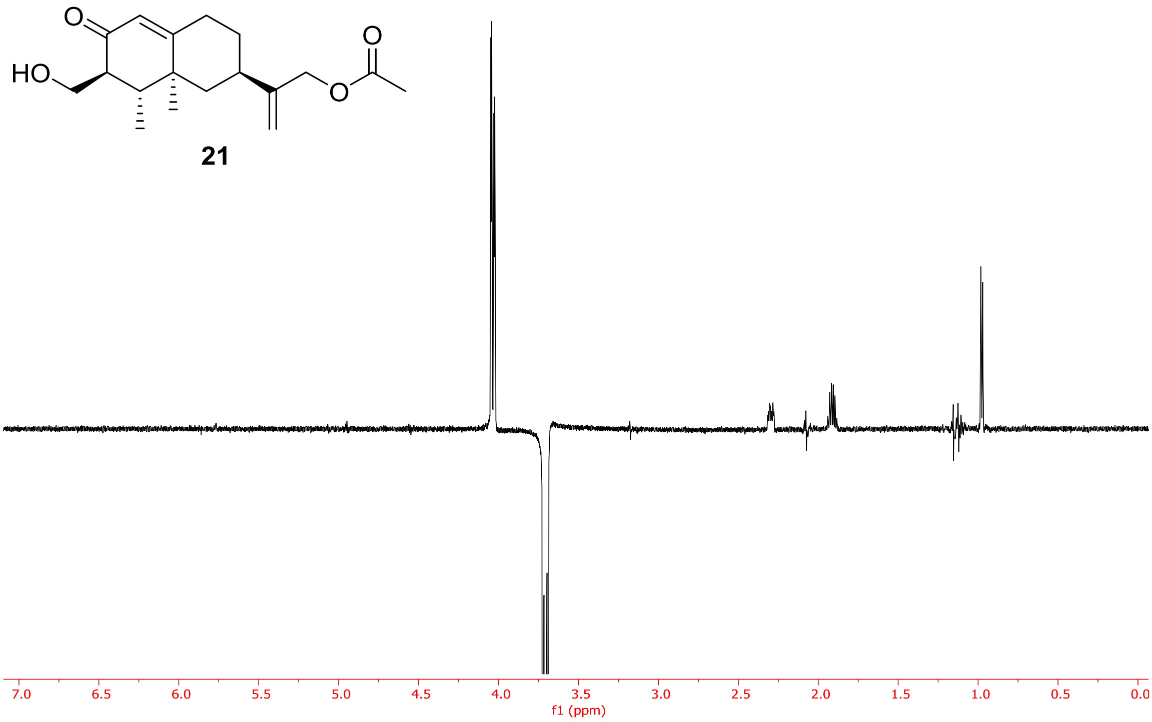
HO
O
O
O
21
7.0 6.5 6.0 5.5 5.0 4.5 4.0 3.5 3.0 2.5 2.0 1.5 1.0 0.5 0.0
f1 (ppm)

NOESY

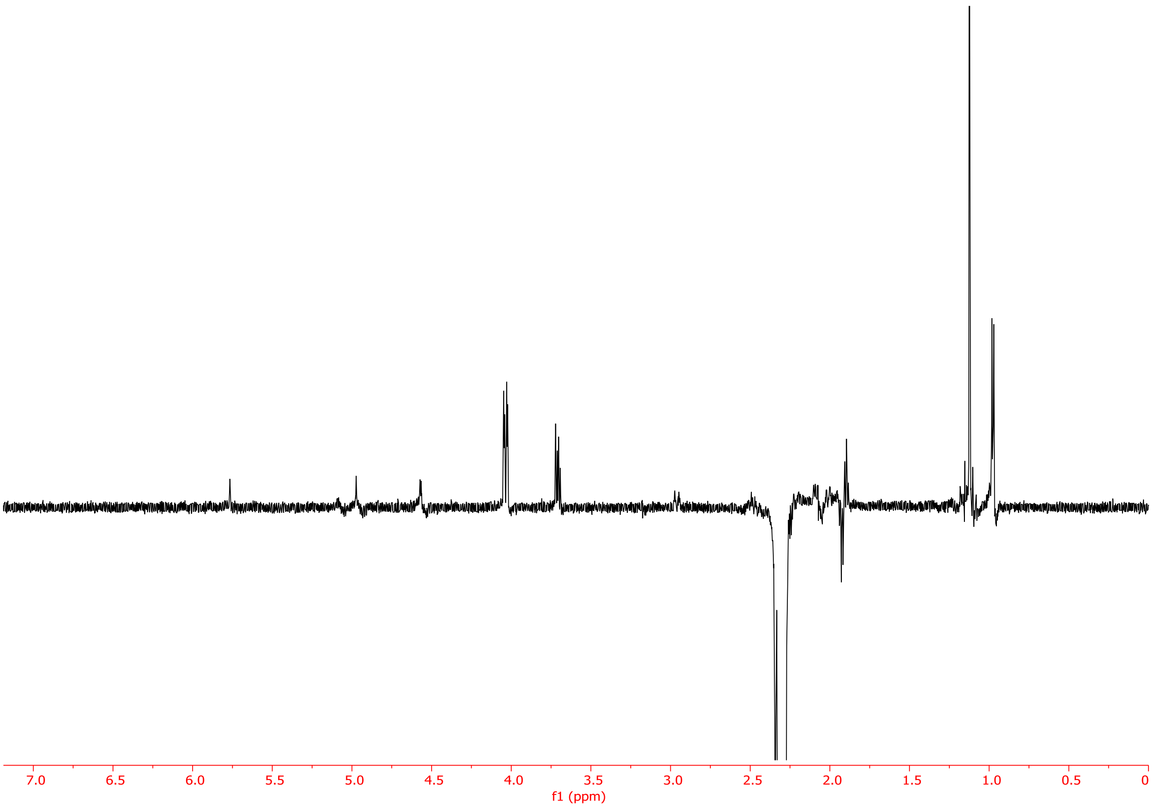
7.0 6.5 6.0 5.5 5.0 4.5 4.0 3.5 3.0 2.5 2.0 1.5 1.0 0.5 0
f1 (ppm)

NOESY

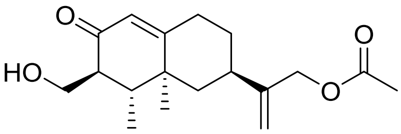

21

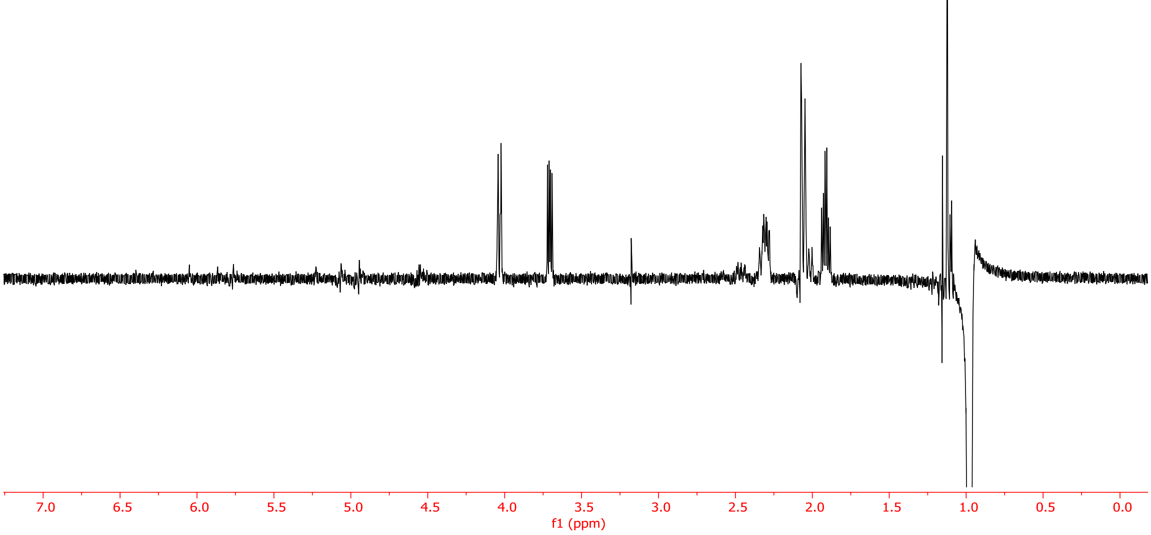

NOESY

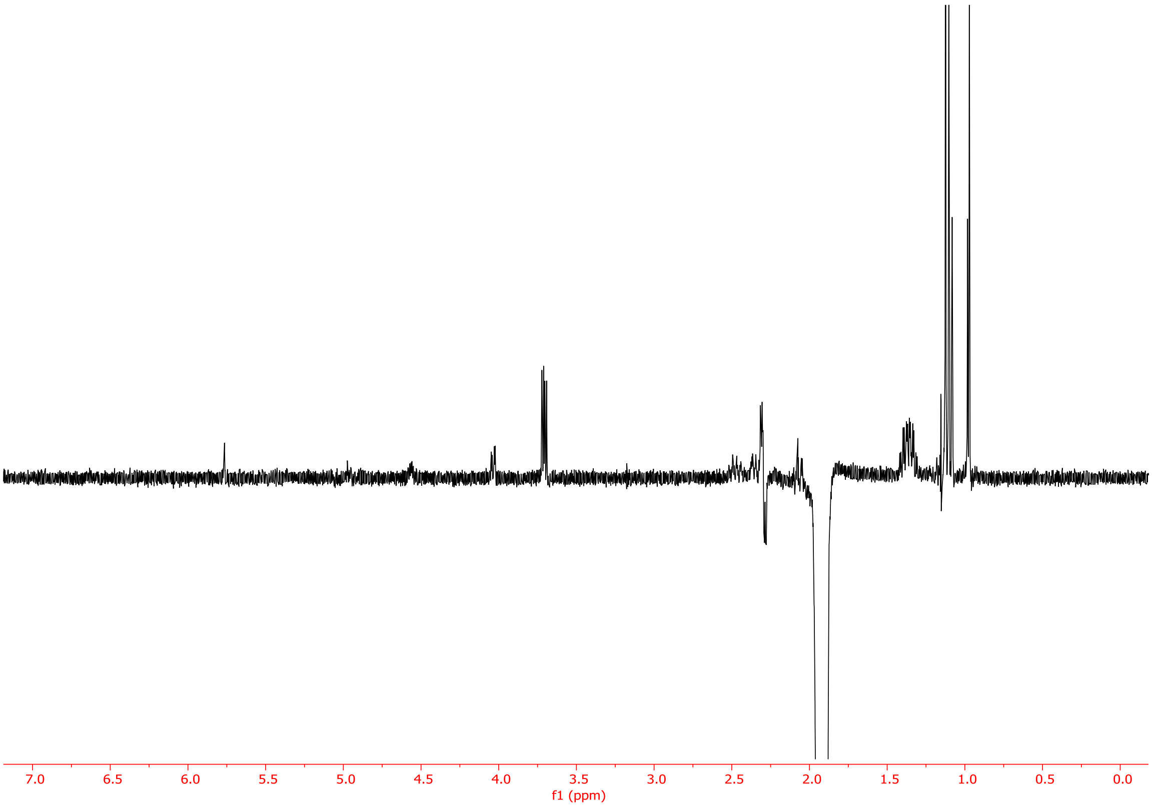

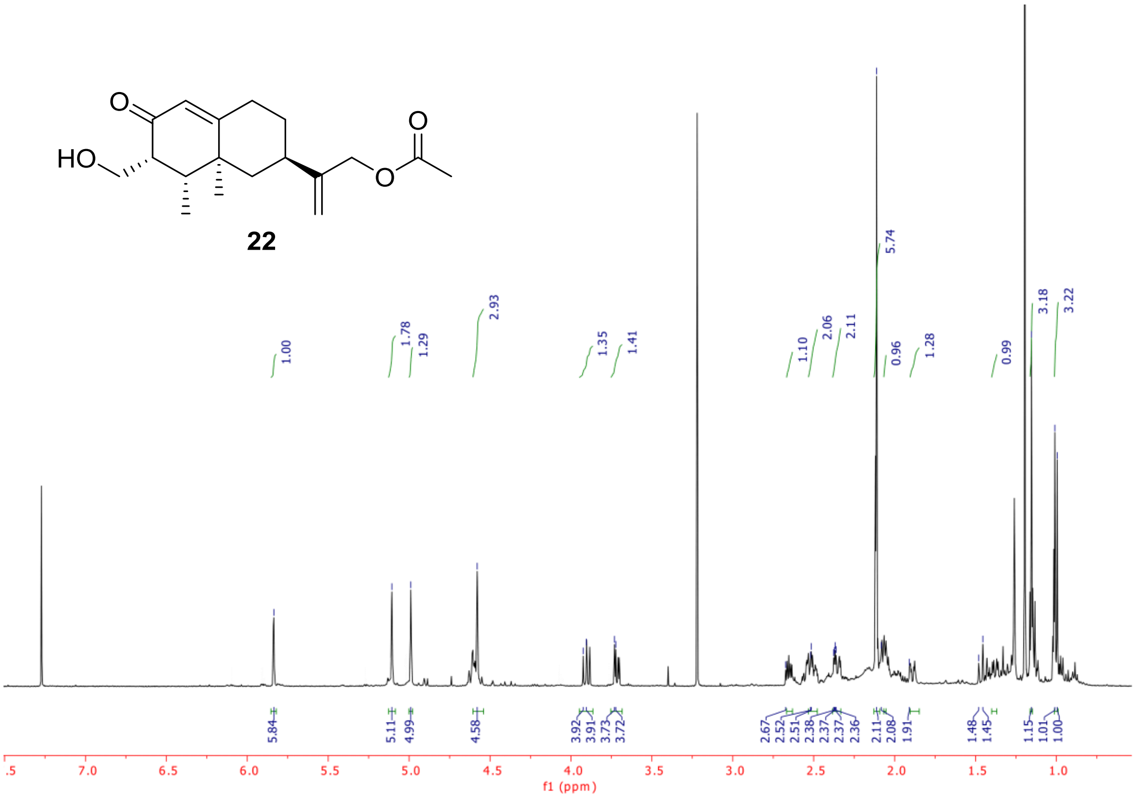
O
HO
O
O
22
1.00
1.78
1.29
2.93
1.35
1.41
1.10
2.06
2.11
5.74
0.96
1.28
0.99
3.18
3.22
5.84
5.11
4.99
4.58
3.92
3.91
3.73
3.72
2.67
2.52
2.51
2.38
2.37
2.37
2.36
2.11
2.08
1.91
1.48
1.45
1.15
1.01
1.00
f1 (ppm)

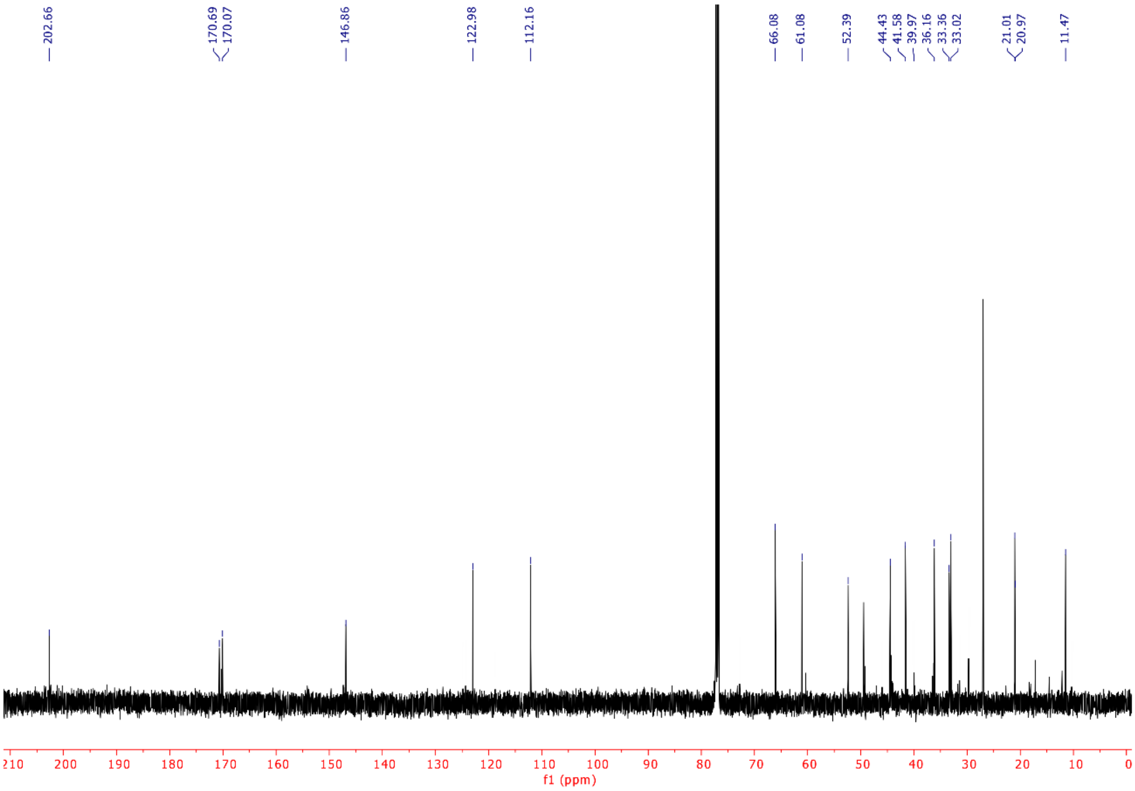
202.66
170.69
170.07
146.86
122.98
112.16
66.08
61.08
52.39
44.43
41.58
39.97
36.16
33.36
33.02
21.01
20.97
11.47
f1 (ppm)

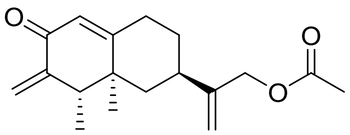

23

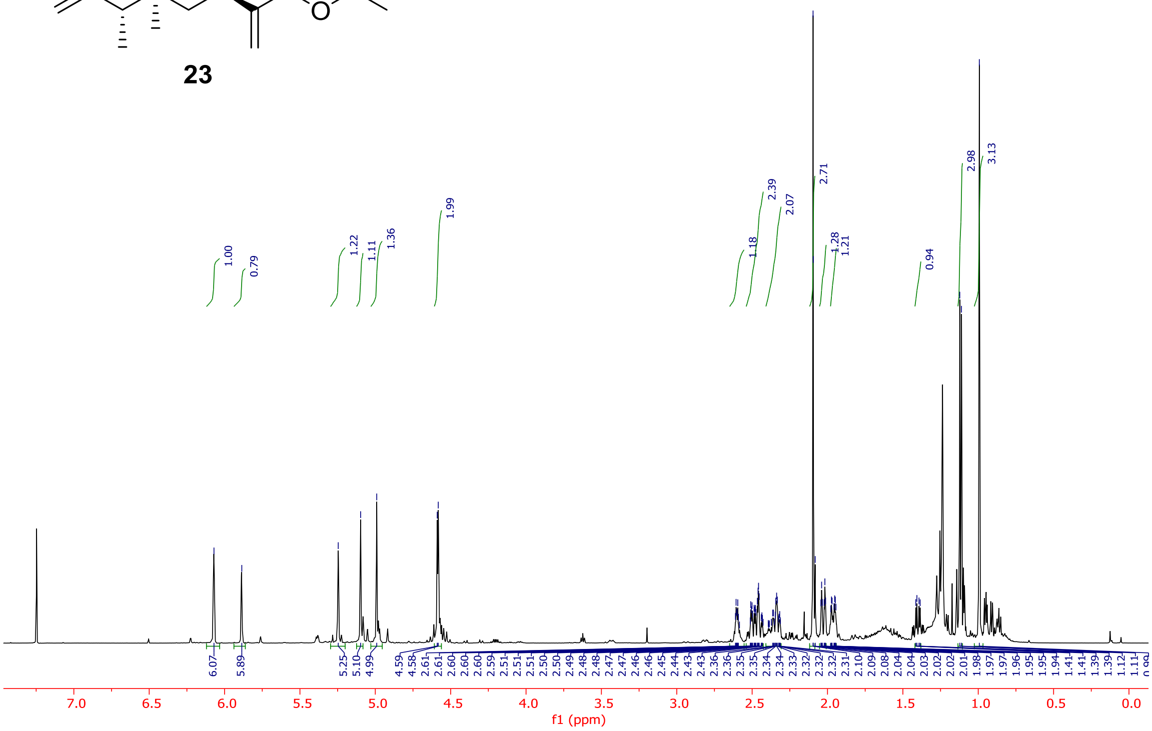

1.00
0.79
1.22
1.11
1.36
1.99
1.18
2.39
2.07
2.71
1.28
1.21
0.94
2.98
3.13
6.07
5.89
5.25
5.10
4.99
7.0 6.5 6.0 5.5 5.0 4.5 4.0 3.5 3.0 2.5 2.0 1.5 1.0 0.5 0.0
f1 (ppm)

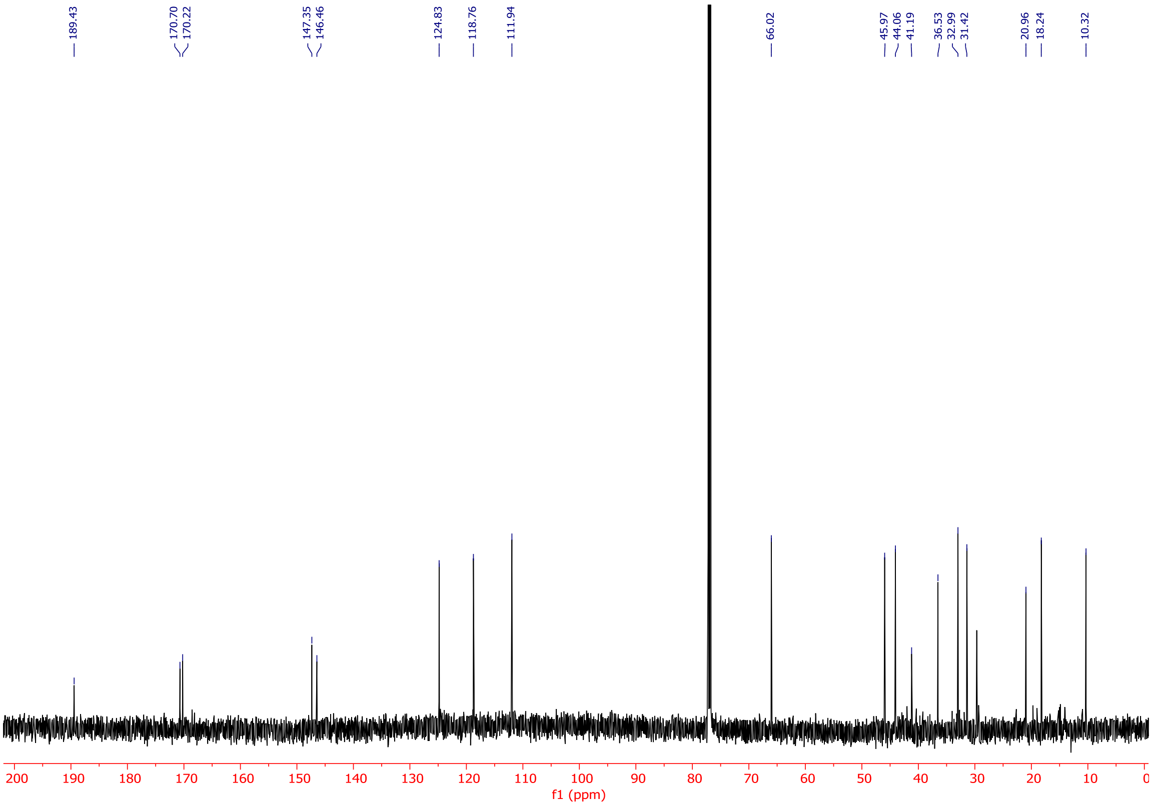

189.43
170.70
170.22
147.35
146.46
124.83
118.76
111.94
66.02
45.97
44.06
41.19
36.53
32.99
31.42
20.96
18.24
10.32
200 190 180 170 160 150 140 130 120 110 100 90 80 70 60 50 40 30 20 10 0
f1 (ppm)
